# Wnt5a promotes hippocampal postsynaptic development and GluN2B-induced expression via the eIF2α HRI kinase

**DOI:** 10.1038/s41598-021-86708-y

**Published:** 2021-04-01

**Authors:** Eva Ramos-Fernández, Macarena S. Arrázola, Carolina A. Oliva, Sebastián B. Arredondo, Lorena Varela-Nallar, Nibaldo C. Inestrosa

**Affiliations:** 1 Centro de Envejecimiento y Regeneración (CARE UC), CARE UC Biomedical Center, Departamento de Biología Celular y Molecular, Facultad de Ciencias Biológicas, Pontificia Universidad Católica de Chile, Av. Alameda 340, 8331150 Santiago, Chile; 2 Instituto de Ciencias Biomédicas, Facultad de Medicina y Facultad de Ciencias de La Vida, Universidad Andrés Bello, Santiago, Chile; 3 Centro de Excelencia en Biomedicina de Magallanes (CEBIMA), Universidad de Magallanes, Punta Arenas, Chile; 4 Present Address: École polytechnique fédérale de Lausanne, Lausanne, Switzerland; 5 Present Address: Centro de Biología Integrativa, Universidad Mayor, Santiago, Chile

**Keywords:** Biochemistry, Cell biology, Molecular biology, Neuroscience, Molecular medicine, Neurology

## Abstract

Wnt signaling plays a key role in neurodevelopment and neuronal maturation. Specifically, Wnt5a stimulates postsynaptic assemblies, increases glutamatergic neurotransmission and, through calcium signaling, generates nitric oxide (NO). Trying to unveil the molecular pathway triggering these postsynaptic effects, we found that Wnt5a treatment induces a time-dependent increases in the length of the postsynaptic density (PSD), elicits novel synaptic contacts and facilitates F-actin flow both in in vitro and ex vivo models. These effects were partially abolished by the inhibition of the Heme-regulated eukaryotic initiation factor 2α (HRI) kinase, a kinase which phosphorylates the initiation translational factor eIF2α. When phosphorylated, eIF2α normally avoids the translation of proteins not needed during stress conditions, in order to avoid unnecessary energetic expenses. However, phosphorylated eIF2α promotes the translation of some proteins with more than one open reading frame in its 5′ untranslated region. One of these proteins targeted by Wnt-HRI-eIF2α mediated translation is the GluN2B subunit of the NMDA receptor. The identified increase in GluN2B expression correlated with increased NMDA receptor function. Considering that NMDA receptors are crucial for excitatory synaptic transmission, the molecular pathway described here contributes to the understanding of the fast and plastic translational mechanisms activated during learning and memory processes.

## Introduction

Wnt ligands are secreted proteins that play a key role in the formation and function of synapses in both developing and mature brain^1–3^. Diverse Wnt ligands are expressed in the central nervous system and activate different Wnt signaling pathways^4,5^. Most studies indicate that Wnt5a primarily activates the non-canonical Wnt pathways at postsynaptic terminals to improve synaptic function^6,7^. For instance, Wnt5a increases the clustering but not the expression of postsynaptic density protein-95 (PSD-95) at the postsynaptic terminal in dendritic spines through the redistribution of an existing PSD-95 pool, an effect mediated by the activation of the Wnt/JNK signaling pathway^8^. Wnt5a also increases the insertion of glutamate receptors at the cell surface^9,10^ and upregulates synaptic N-methyl-D-aspartate receptor (NMDAR) currents in the hippocampus, stimulating the induction of long-term potentiation (LTP)^11^. Besides, several evidences show that in cultured hippocampal neurons Wnt5a-induced activation of Wnt/Ca^2+^ signaling activates dendritic spine morphogenesis, de novo formation of spines and increases the size of preexisting spines^10,12–14^. In vivo, the chronic infusion of Wnt5a also induces the increase of perforated synapses, associated with synaptic potentiation^15^. In addition to the role of Wnt5a in excitatory synapses, Wnt5a can also regulate inhibitory synapses through the insertion and recycling of ionotropic gamma-aminobutyric acid receptors (GABA_A_Rs) by the activation of calcium/calmodulin-dependent protein kinase II (CaMKII)^16^. Despite all of this information, no reported studies assess the ultrastructural changes in dendritic spines mediated by Wnt5a, or the mechanisms involved.

Previously, we reported that in hippocampal neurons Wnt5a induces nitric oxide (NO) production through a mechanism depending on intracellular calcium levels, and NO increases the trafficking of NMDAR-containing vesicles to the postsynaptic membrane^17^. NO acts as a messenger to modulate neuronal function^18^, increasing the release of various neurotransmitters^19^, including glutamate^20^. NO has been implicated in the regulation of neuronal excitability during LTP, as well as in memory processes^17,18^. Among the effects of NO, the activation of the heme-regulated eukaryotic initiation factor eIF2α (HRI) kinase has been described as crucial during the consolidation of object recognition memory^21^. The HRI kinase is present in synaptosomes and colocalizes with PSD-95^22,23^. The activation of HRI kinase triggers the translation of mRNAs with a long 5′ untranslated region (5′UTR) and several upstream open reading frames (uORFs), contributing to spine growth, synaptogenesis and the consolidation of hippocampal memory^23^. We previously showed that through NO production, glutamate can stimulate HRI and the translation of the GluN2B subunit of NMDAR^22^.

Here, we report that Wnt5a induces an increase in spine density of hippocampal neurons, concomitant with a time-dependent increase in the length and area of the PSD of dendritic spines in mouse brain slices. Ultrastructural analysis showed that after this spine remodeling, Wnt5a induces the formation of perforated PSDs and new dendritic spines. The expression of the GluN2B subunit increases upon short treatments with Wnt5a, and its function is also enhanced. Finally, we assessed the involvement of HRI kinase in these effects, and determined that the inhibition of HRI kinase activity, both pharmacologically and genetically, abolished all the effects of Wnt5a. These results reveal a novel Wnt5a/ HRI kinase pathway involved in synapse formation and remodeling.

## Results

### Wnt5a induced a time-dependent increase in the length of the PSD and synaptic contacts in the hippocampal CA1

**Figure 1:**
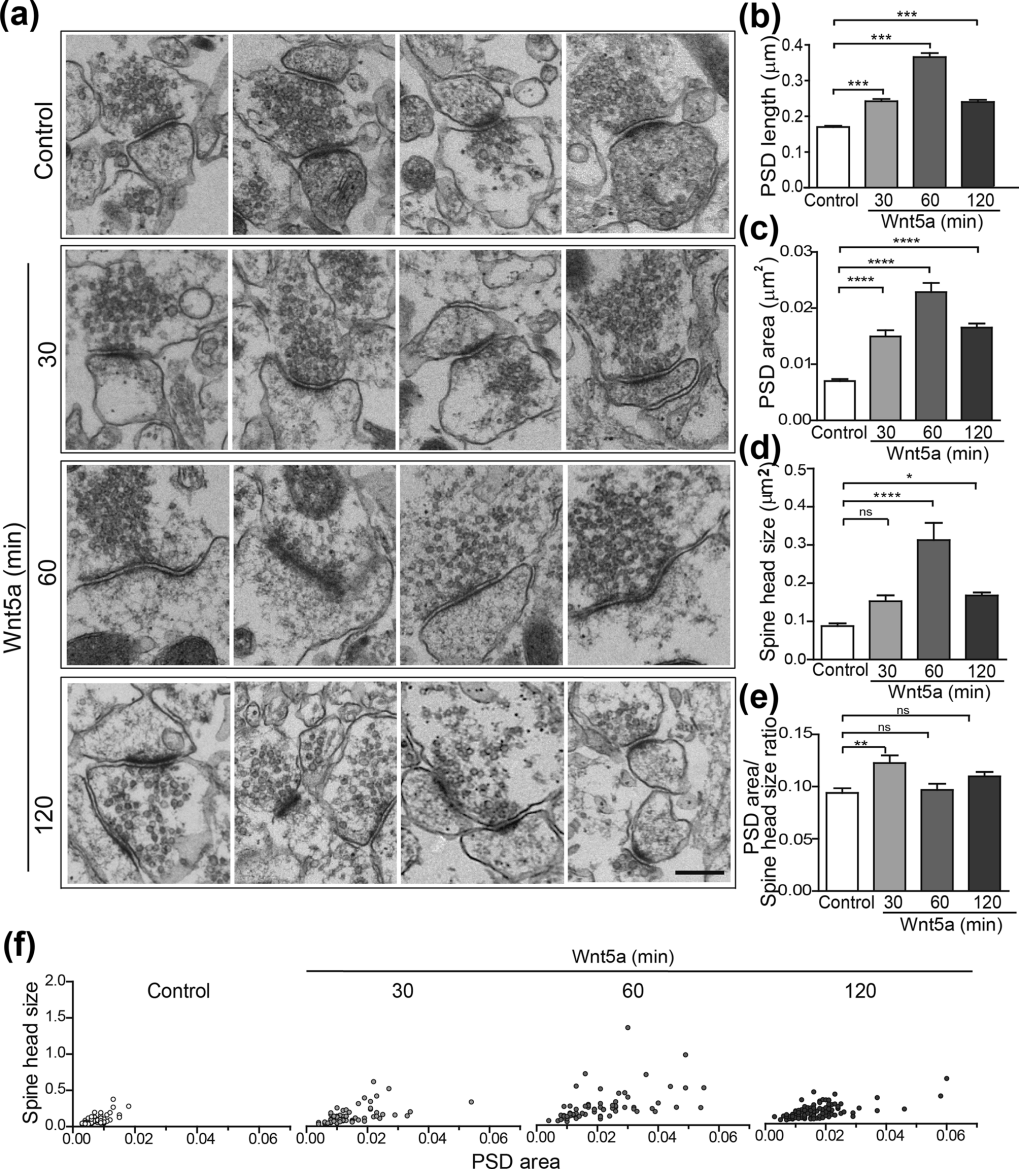
Wnt5a increases the PSD length in hippocampal slices. (**a**) EM images from the CA1 region of hippocampal slices exposed to Wnt5a conditioned medium for 30, 60 and 120 min. Scale bar = 250 nm. We include 4 different images for each condition. Wnt5a induced a time-dependent increase in the average PSD length (**b**) and area (**c**) in dendritic spines, with a reduction at 2 h. The spine head size was increased after 30 to 120 min of Wnt5a exposure (**d**) and the PSD area/spine size ratio (**e**) was induced at 30 min of Wnt5a treatment. N = 3 independent animals; Control (368); 30 (291); 60(247); 120(371) synaptic contacts. (***p = 0.00009).

First, before the experiments, we measured the presence of Wnt5a on conditioned medium, we tested its ability to activate the non-canonical Wnt pathway and its toxicity (Supplementary Fig. S1a c,d). To test the effect of Wnt5a in the formation of spines, primary hippocampal neurons transfected with EGFP were treated with Wnt5a for 1 h. An increase in the density of dendritic spines was observed in neurons after treatment with Wnt5a as has been reported previously (Supplementary Fig. S2a)^10^. Then, we performed electron microscopy (EM) on slices exposed to Wnt5a for different incubation times to assess structural changes in dendritic spines and synaptic contacts. The first structural change observed in spines by the effect of Wnt5a was the enlargement of the PSDs (Fig. 1a). The PSD length is a measure of the synaptic contact size and was analyzed as previously described^24^. The analysis indicates that the PSD length increases by the effect of Wnt5a at 30, 60 and 120 min, reaching a maximum length of 1.22 μm at 60 min (Fig. 1b and Table 1). This effect occurred in the entire population of the spines, rather than in a fraction of them, and was independent of the initial length of the spines (Table 1). The PSD area (Fig. 1c) mimic the results obtained for the PSD length. Wnt5a increases the PSD area over time showing the highest increase after 60 min of treatment. Interestingly, the spine head size was also significantly increased at 60 and 120 min of Wnt5a exposure compared with the control condition (Fig. 1d). When analyzing the PSD area/spine head size ratio, a significant increase was observed only at 30 min of Wnt5a treatment (Fig. 1e). This result shows that Wnt5a first modifies the PSD structure and then the spine head size. The correlation between the spine head size and the PSD area (Fig. 1f) shows that Wnt5a mainly induced changes in the PSD area independently of the spine head size. After 60 min of treatment there is a bigger dispersion of the data since both, PSD area and spine head size are increased. Finally, at 120 min the dispersion is more similar to the 30 min, but the effect on PSD area/spine size ratio is not significant indicating that both parameters were similarly increased. These results indicate that Wnt5a first effects appear at the PSD region and then the restructuring of the spine size occurs.

**Table 1:** Percentile values and statistical analysis of the length of post-synaptic density from EM images.

	Control	Wnt5a (min)		
		30	60	120
Number of values	**368**	**291**	**247**	**371**
Minimum	0.061	0.061	0.074	0.0739
25% Percentile	0.116	0.163	0.2345	0.1587
		^A^(+40.5)	^A^(+102.2)	^A^(+36.8)
Median	0.1535	0.228	0.33	0.2152
75% Percentile	0.212	0.307	0.447	0.296
		^A^(+44.8)	^A^(+110.8)	^A^(+39.6)
Maximum	0.443	0.653	1.22	0.9759
Mean	0.1701	0.2423	0.3659	0.2401
		^A^(+42.4)	^A^(+115.1)	^A^(+41.2)
SD	0.07218	0.1073	0.1812	0.1203
Std. Error of Mean	0.003762	0.006291	0.01153	0.006246
Lower 95% CI	0.1627	0.2299	0.3432	0.2278
Upper 95% CI	0.1775	0.2547	0.3886	0.2524

PSD length values grouped by percentile for each treatment. The values are represented in micrometers^2^ (µm^2^) and “A” corresponds to the percentage of increase in PSD length of each group when compared to the corresponding Control group. The significance of bold (Number of values) indicates the number of dendritic spines analyzed for each treatment.

**Figure 2:**
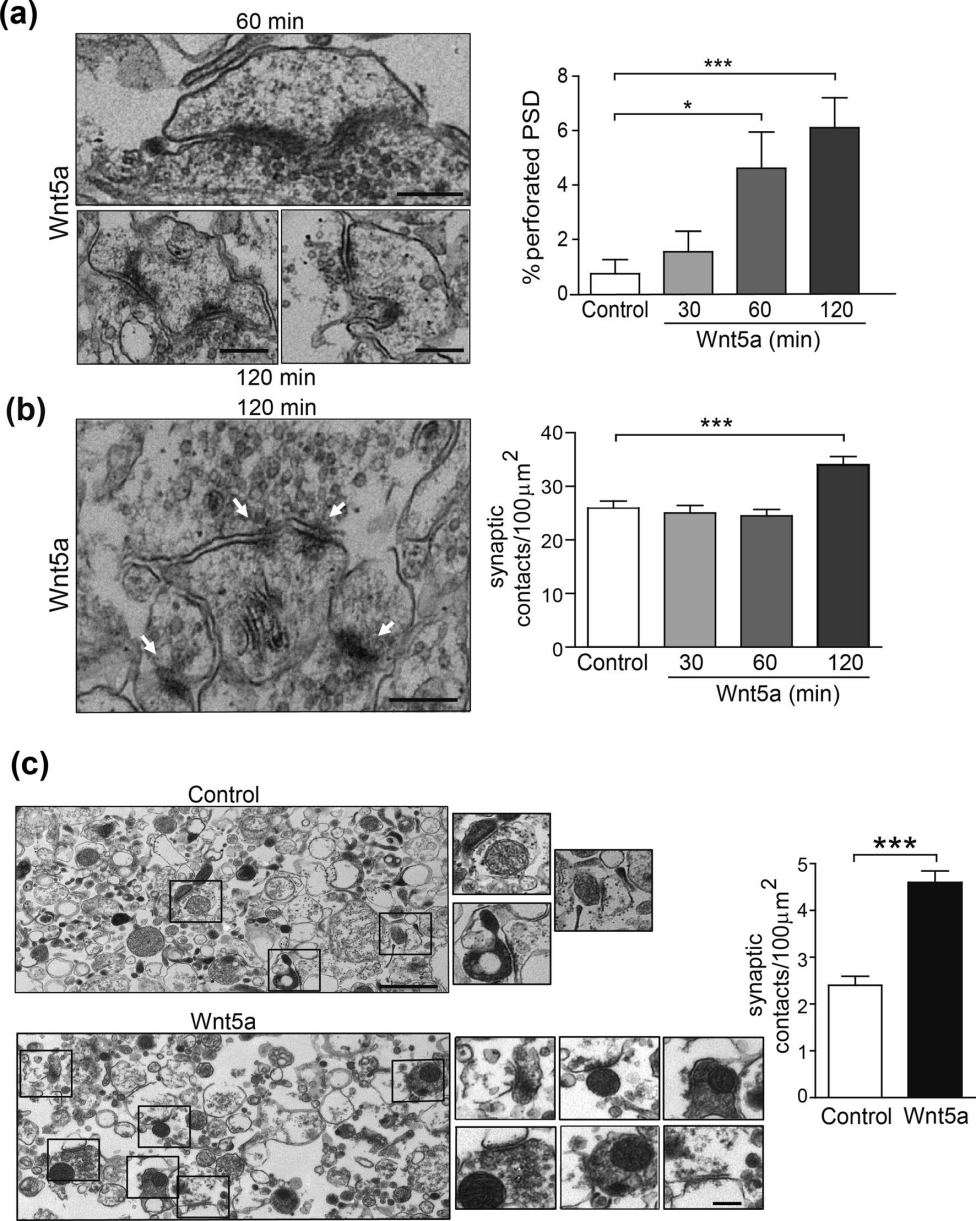
Wnt5a stimulation increases the number of spines with perforated PSD and synaptic contacts in hippocampal slices and synaptosomes. Representative EM images of perforated PSDs from the CA1 region of hippocampal slices (**a**) and multiple synapses (**b**), in response to 2 h of treatment with Wnt5a. Quantification of the time-course effect of Wnt5a shows an increased number of synaptic contacts per 100 μm^2^ area at 2 h (**b**) and a time-dependent increase in the percentage of dendritic spines presenting segmented PSD (a). Scale bar: 250 nm. N = 3 independent animals; Control (61); 30 (51); 60(45); 120 (48) photos (*p  0.05, ***p < 0.0001). (**c**) Synaptosome fraction isolated from mouse brain and treated for 1 h with Wnt5a. Representative EM images showing the number of PSDs and the number of multiple synaptic contacts after treatment with control or Wnt5a conditioned medium and the graph bar showing the quantifications. N = 3 independent animals Control (50); Wnt5a (59) photos (***p < 0.0001). Scale bars: 1 μm and 250 nm, respectively.

At 1 h of Wnt5a treatment there was also an increase in the percentage of spines with segmented PSDs (or perforated synapses) in slices. Strikingly, this effect was still observed after 2 h of treatment, where spines with perforated PSD reached a fivefold increase (Fig. 2a and Supplementary Fig. S2b). Therefore, sequentially, the treatment of hippocampal slices with Wnt5a produces an increase in PSD length and area that begins at 30 min to 1 h; then, the percentage of spines with segmented PSDs also increased, being more pronounced at 2 h. In agreement with this effect, after 2 h the PSD length was reduced (Fig. 1b), but the number of synaptic contacts was increased (Fig. 2b). Using synaptosomes isolated from the mouse brain, we observed the same effect in the number of synaptic contacts (Fig. 2c). These findings suggest that the spines with segmented PSDs had split into new synaptic contacts, and therefore, new spines were formed.

Altogether, these results demonstrate that Wnt5a modifies the structure of dendritic protrusions and induces the generation of new mature spines.

### Wnt5a-NO signaling induced HRI activation and GluN2B expression

In previous work we determined that Wnt5a induces the production of NO in hippocampal neurons^17^. Here, by using DAF-FM probe (a dye that binds NO) we confirmed that Wnt5a induces NO and that the inhibition of NOS with 7-NI abolish that effect (Supplementary Fig. S6). NO is known to activate HRI kinase^25^, one of the four kinases that can phosphorylate the initiation factor eIF2α and that colocalizes postsinaptically with PSD-95^23^. We evaluated whether the activation of HRI kinase is a downstream effector of Wnt5a in the induction of spine remodeling.

**Figure 3:**
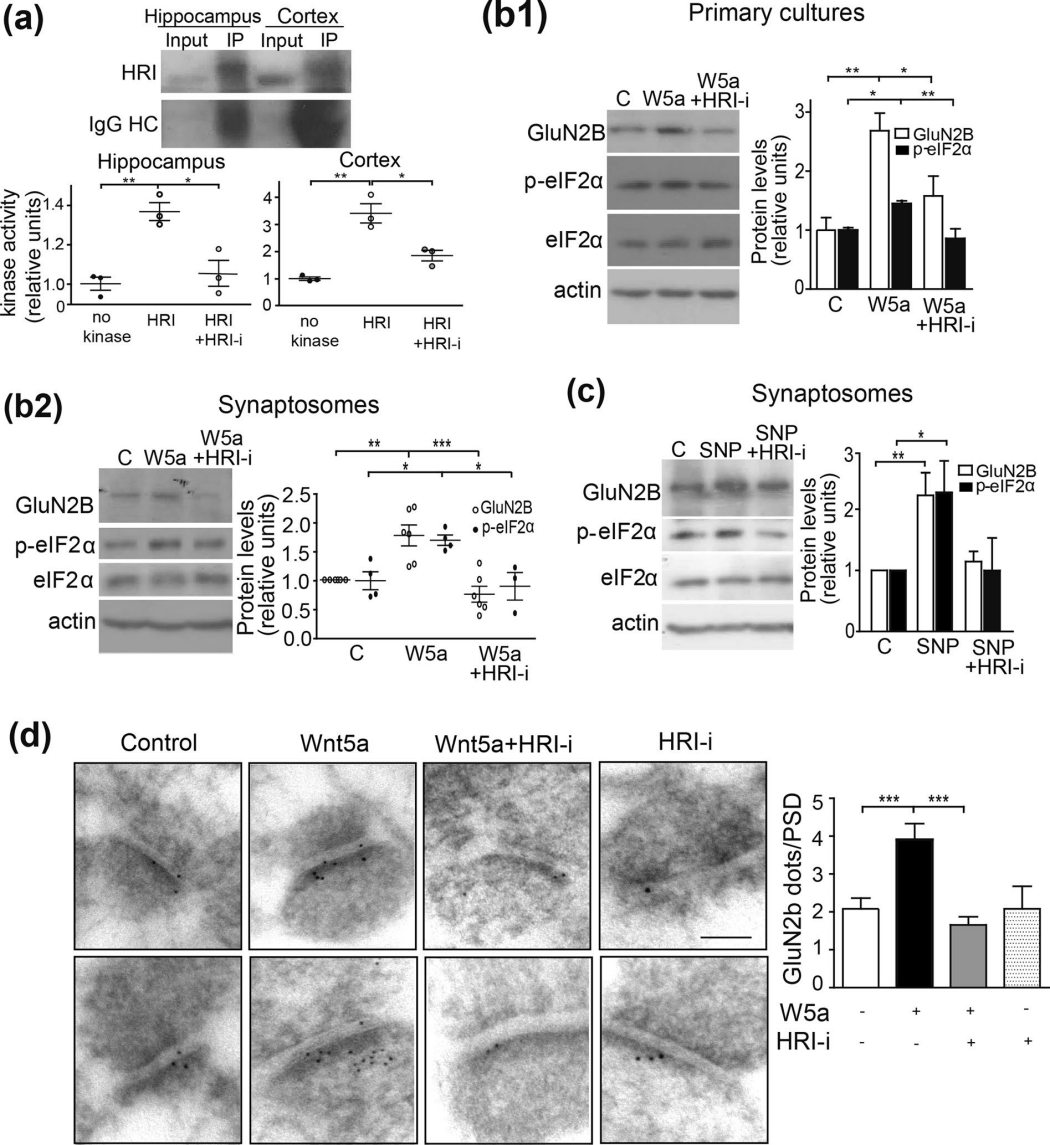
Wnt5a mediates GluN2B expression through HRI kinase activation. (**a**) Blot showing immunoprecipitated HRI kinase from mouse hippocampus and cortex. Graphs at bottom show the fold kinase activity of the immunoprecipitated kinase in the presence or absence of the HRI inhibitor, measured by luciferase signal. Lines show the mean ± SEM. N = 3 independent animals. (*p  0.05, **p < 0.001) *p* = 0.0041 (hippocampus) *p* = 0.001 (cortex). (**b**) Western blots of primary cultures (b_1_) or synaptosomes (b_2_) (**c**) after treatment with control, Wnt5a (W5a) medium or nitric oxide donor sodium nitroprusside (SNP) or co-incubation with Wnt5a, SNP and HRI-i. N = 8 (GluN2B)-7 (eiF2α) independent experiments (primary cultures), N = 6 (GluN2B)—4 (eiF2α) independent experiments (Wnt5a treated synaptosomes) and N = 5 (GluN2B)—7 (eiF2α) respectively ±SEM. (*p  0.05, **p < 0.001, ***p < 0.90005), *p* = 0.0021 (GluN2B in primary cultures): *p* = 0.0004 (eIF2α in primary cultures); *p* = 0.0003(GluN2B in Wnt5a treated synaptomes) ; *p* = 0.0131(eIF2α in Wnt5a treated synaptosomes); *p* = 0.0108 (GluN2B in SNP treated synaptomes); *p* = 0.0531 (eIF2α in SNP treated synaptosomes). (**d**) EM images from the CA1 region of hippocampal slices with immunogold against GluN2B subunit. Graph shows quantification of the gold particles in the postsynaptic area. Scale bar: 100 nm. Lines show the mean ± SEM. N = 3 independent animals; Control (85); Wnt5a (76); Wnt5a+HRI-i (107); HRI-i (24) photos (***p = 0.00009). Full-length gels are presented in Supplementary Fig. S3A.

Previously, we determined that HRI kinase is active in the cortex^22^. Here, we also showed the presence of its kinase activity in the hippocampus (Fig. 3a and Supplementary Fig. S3Aa), and that its activity was inhibited by the specific inhibitor HRI-i^26^. We observed a complete inhibition of its activity (100%) in the immunoprecipitated kinase from the hippocampus, and an 80% reduction in the immunoprecipitated kinase from the cortex (Fig. 3a), confirming the reliability of this inhibitor. On the other hand, it is known that translation of GluN2B subunit of NMDAR is regulated by NO production and the phosphorylation of eIF2α^22^. The phosphorylation of eIF2α has been associated with inhibition of the translation of major proteins; however, the translation of proteins with long 5′UTRs and more than one initiation codon (AUG) is enhanced. One example of such proteins is the GluN2B subunit, which contains a 179 bp 5′UTR with 3 upstream AUGs. Considering this, we evaluated the effect of Wnt5a on the expression of GluN2B in primary cultured neurons or synaptosomes treated 1 h with control or Wnt5a conditioned medium. The Wnt5a treatment significantly increased GluN2B levels both in primary cultures (Fig. 3b1) and in synaptosomes (Fig. 3b2 and Supplementary Fig. S3Ab_2_). This increase in GluN2B levels correlated with increased phosphorylation of eIF2α on serine 51 (S51), the substrate of HRI kinase (Fig. 3b1,b2), and both effects were blocked by co-treatment with HRI-i (Fig. 3b1,b2). These effects of Wnt5a were mimicked by treatment of synaptosomes with sodium nitroprussiate (SNP), a NO donor (Fig. 3c and Supplementary Fig. S6).

The effect of Wnt5a treatment was also evaluated by immunogold detection assay, which showed an increase in GluN2B subunits specifically localized in the postsynaptic region after 1 h of Wnt5a treatment (Fig. 3d and Supplementary Fig. S3B). This increase was not observed in the presence of HRI-i (Fig. 3d). Altogether these findings show that Wnt5a promotes eIF2α phosphorylation and GluN2B expression through activation of HRI kinase.

### GluN2B translation is de-repressed by Wnt5a

**Figure 4:**
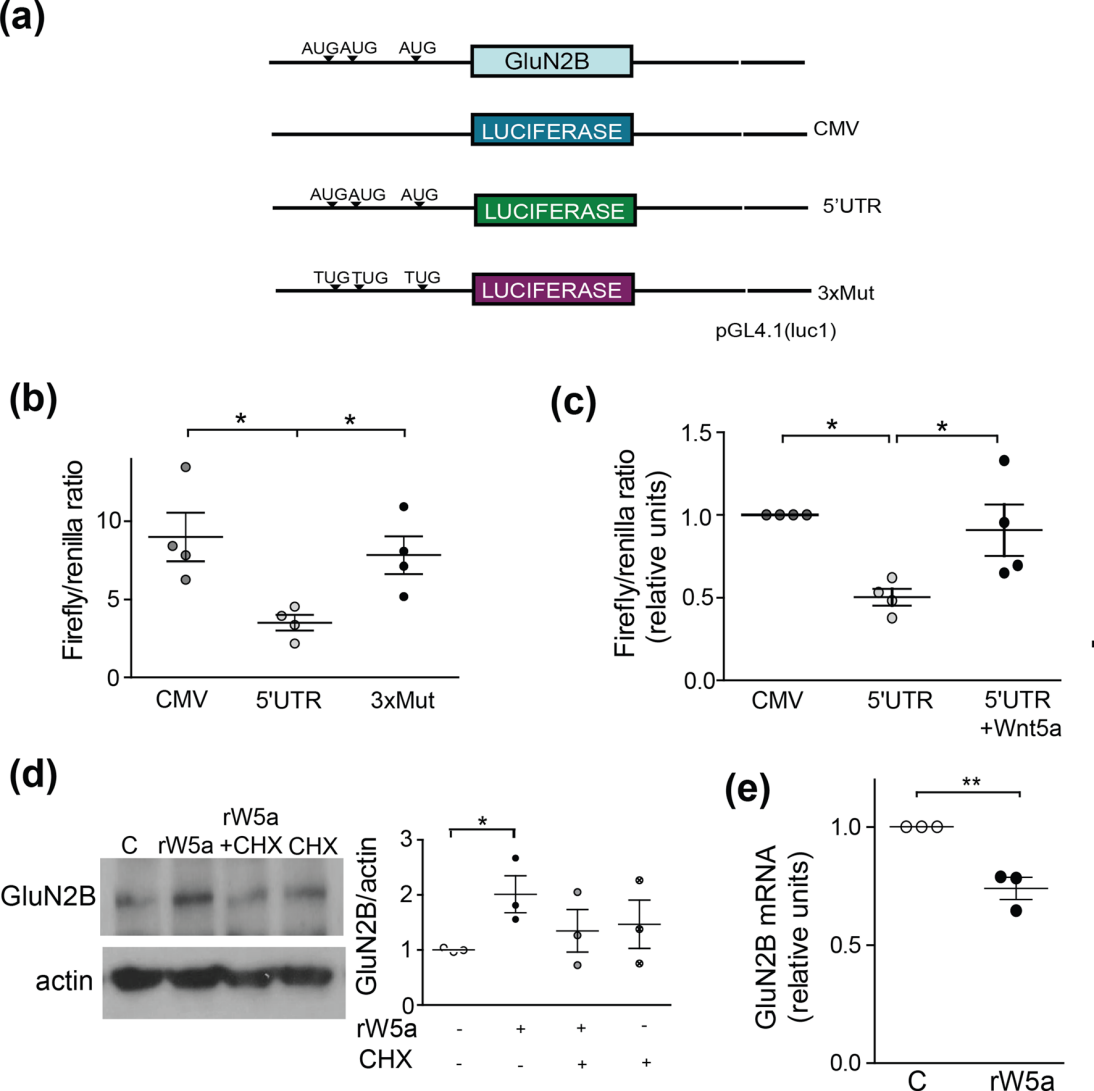
5′UTR-GluN2B translational de-repression by Wnt5a ligand (**a**) Scheme depicting the 5′UTR of the GluN2B subunit (179 bp and 3 upstream AUGs), and the luciferase constructs designed for the reporter assay. CMV, contains luciferase gene under CMV promoter; 5′UTR, contains 5′UTR from GluN2B cloned between the CMV promoter and luciferase gene; 3xMut, contains the 5′UTR with the three uAUGs mutated to uTUGs. (**b**) Luciferase assay in HT22 cells transfected with the 3 reporter vectors. Lines show the mean ± SEM. N = 4 independent experiments (*p = 0.00.41; *p* = 0.0157) (**c**) Luciferase assay in hippocampal primary neurons transfected with the vectors and treated with Wnt5a, expressed as relative units. Lines represent mean ± SEM. N = 4 (*p = 0.0011; *p* = 0.0288; *p* = 0.0245) (**d**) Representative blot showing hippocampal neurons treated with 300 ng/mL recombinant Wnt5a (rW5a), rW5a+CHX or CHX. The plot shows the GluN2B expression relative to actin levels. Lines show the mean ±SEM.N=3 independent experiments. Dunnett’s Multiple comparison test (*p = 0.0315). (**e**) qRT-PCR from total RNA isolated from hippocampal neurons after 1 h of not treated or treatment with rW5a. GluN2B mRNA levels were normalized to GAPDH mRNA and expressed relative to control condition. Lines represent mean ± SEM. N = 3 independent experiments (**p = 0.0051). Full-length blots are presented in Supplementary Fig. S4.

We previously reported that the 5′UTR of GluN2B regulates its translation by repressing it. Instead, under NO stimulus it can be de-repressed, as has been shown in the cell line SH-SY5Y^22^. Therefore, we designed 3 different reporter constructs with or without the wild-type or mutated 5′UTR of the GluN2B subunit (Fig. 4a). This region is 179 bp and contains three upstream AUGs that we cloned into a pGL4.1 (luc1) vector downstream of the CMV promoter to regulate the luciferase reporter expression. As a control, we generated a vector with the same structure without the 5′UTR or with the 5′UTR mutated in the three upstream AUGs (a conservative mutation of AUG to TUG). The co-transfection of these constructs with a *Renilla* luciferase vector into HT22 cells resulted in the same effect observed in SH-SY5Y cells. The 5′UTR from GluN2B decreased the *Firefly*/*Renilla* luciferase ratio compared with the CMV and mutated constructs (Fig. 4b). In primary neurons, the 5′UTR also repressed luciferase expression (Fig. 4c). Interestingly, Wnt5a treatment induced the expression of luciferase in hippocampal neurons transfected with the 5′UTR (Fig. 4c), suggesting that Wnt5a treatment reverted the repression mediated by the GluN2B 5′UTR. Then, considering that the translation of GluN2B is regulated by the phosphorylation of eIF2α, which was induced by Wnt5a treatment (Fig. 3b1,b2), we hypothesized that the effect of Wnt5a on GluN2B expression might depend on translation. Consistent with that, the increase in GluN2B protein level by rWnt5a treatment was not observed in the presence of the translation inhibitor cycloheximide (CHX) (Fig. 4d and Supplementary Fig. S4), strongly suggesting that Wnt5a induces the translation of this NMDAR subunit. Moreover, Wnt5a treatment induced a reduction in GluN2B mRNA levels as assessed by RT-qPCR (Fig. 4e). This indicates that the increase of GluN2B protein levels by Wnt5a treatment is not mediated by an increase in transcription of the GluN2B gene, but by an increase of protein translation.

### HRI knockdown prevented the induction of GluN2B expression by Wnt5a

**Figure 5:**
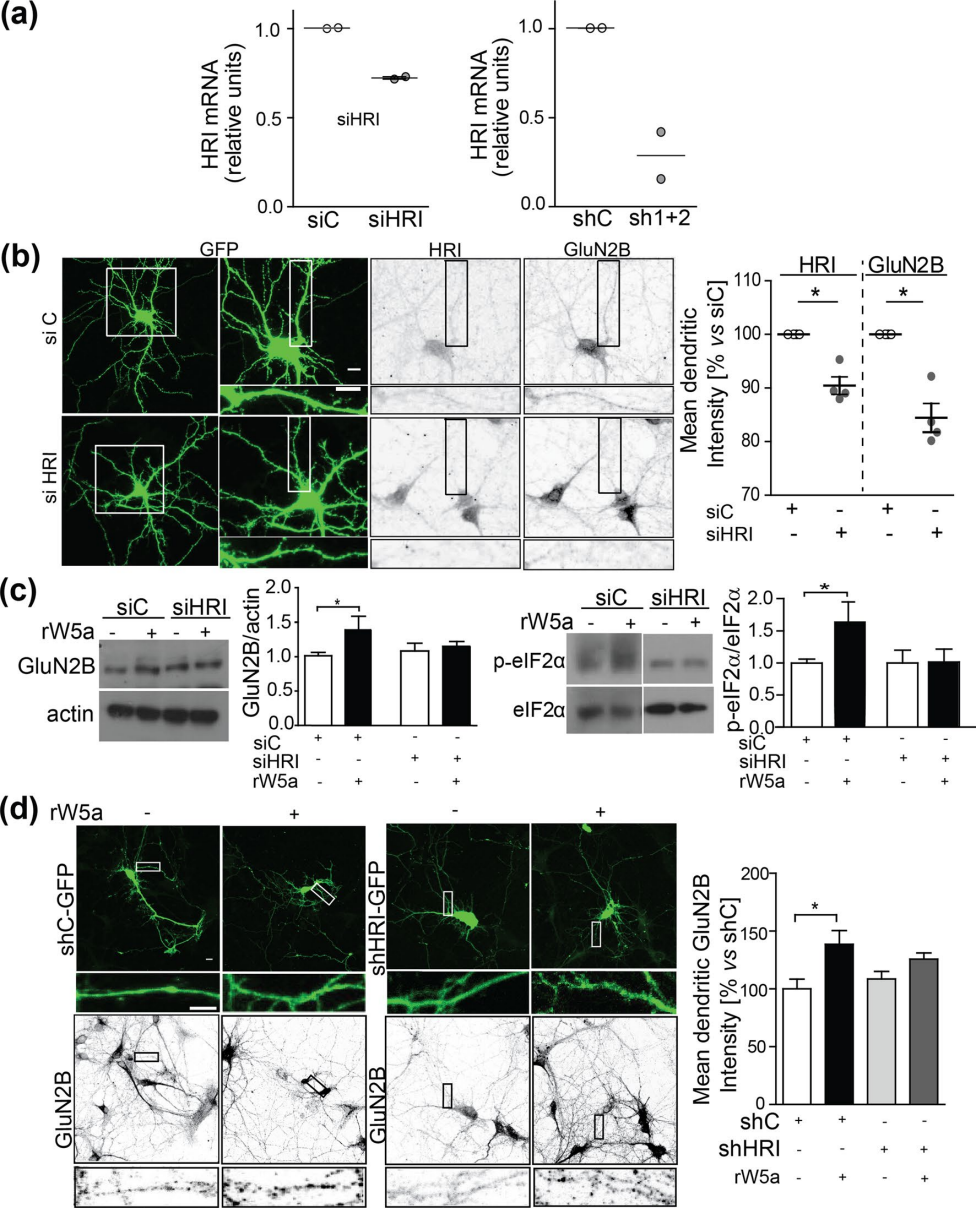
HRI knockdown reduces Wnt5a mediated GluN2B expression. (**a**) qRT-PCR from total RNA isolated from hippocampal neurons after magnetofection with siC and siHRI or infection with shC and sh1+2. HRI mRNA levels were normalized to GAPDH mRNA and are expressed relative to the control condition. Lines show the mean ± SEM N = 2 independent experiments (**b**) Representative images of immunofluorescences from DIV10 hippocampal neurons magnofected at DIV8 with eGFP and siRNA or siHRI, and the respective GluN2B and HRI signals. The right panel show the quantification of N = 4 independent experiments ± SEM (**c**) Representative western blot from hippocampal neurons transfected with siC or siHRI and untreated or treated with 300 ng/mL rWnt5a for 1 h. Graph at right shows the western blot quantifications relative to the control. Bars represent mean ± SEM N = 5. (*p = 0.0254). (**d**) Immunofluorescence detection of the presence of GluN2B in DIV10 hippocampal neurons transfected with bicistronic shC-tGFP or shHRI-tGFP at DIV6-7. Graph quantifying the mean GluN2B intensity in hippocampal dendrites. Bars show the mean ± SEM. N = 3 independent experiments. shC (55); shC+rWnt5a (43); shHRI (115); shHRI+rWnt5a (151) (*p = 0.0069). Full-length blots are presented in Supplementary Fig. S5.

To further evaluate the role of HRI in the effects of Wnt5a, we designed two different approaches to genetically knockdown HRI expression. The first approach was to use a small interfering RNA (siRNA) targeting HRI (siHRI) and a non-targeted siRNA as a control (siC). The second approach was to use a lentivirus expressing turbo GFP (tGFP) and either control (shC) or HRI-targeting (shHRI) short hairpin RNA (shRNA). In hippocampal neurons magnetofected with siHRI or infected with a mix of 2 different shHRI sequences (sh1 + 2), the HRI mRNA levels decreased by ~ 30% or ~ 75% respectively, compared with the controls (Fig. 5a). Once we demonstrated the efficiency of the HRI knockdown, we evaluated whether it affected the expression of HRI and GluN2B in dendrites. Immunofluorescence analysis in neurons co-magnetofected with siRNAs and eGFP, showed that siHRI decreased the HRI and GluN2B subunit levels compared to siC (Fig. 5b). Neurons magnetofected with siC or siHRI were treated at DIV10 with rWnt5a for 1 h. An ~ 50% increase in GluN2B expression in response to Wnt5a was observed in neurons transfected with siC, while no effect was observed in neurons transfected with siHRI (Fig. 5c and Supplementary Fig. S5). Concomitantly, Wnt5a treatment induced an increase in eIF2α phosphorylation that was not observed in neurons magnetofected with siHRI (Fig. 5c). Hippocampal neurons were transfected with shC or shHRI lentiviral vectors and the expression of GluN2B was evaluated by immunofluorescence in cells expressing tGFP (Fig. 5d). GluN2B levels were increased ~ 40% in shC-expressing neurons after 1 h of treatment with rWnt5a, and this effect was not observed in neurons expressing shHRI (Fig. 5d). These results support the hypothesis that HRI is required for Wnt5a-mediated GluN2B expression.

### Wnt5a increased functional NMDARs

**Figure 6:**
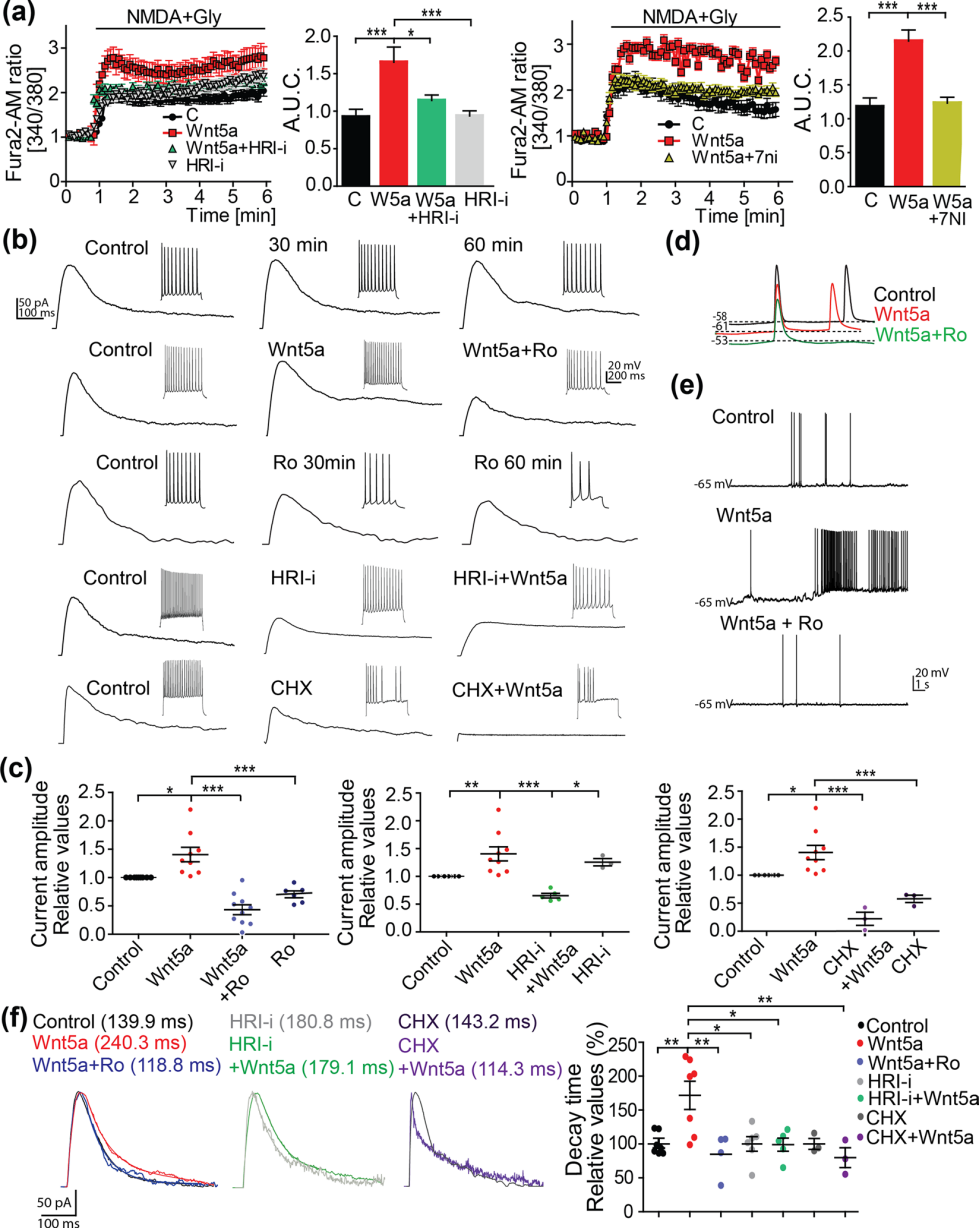
Wnt5a increases the GluN2B-containing NMDAR activity. (**a**) Plots show the normalized Fura-2 AM fluorescence ratio (340/380) over time before and after NMDA + glycine exposure in neurons treated for 1 h with control (C) or Wnt5a (W5a) medium in the presence or absence of HRI-i and 7-NI. Graphs show the AUC of the represented plots. Bars show the mean ± SEM. N = 6 independent experiments. Control (100); Wnt5a (110); Wnt5a+HRI-I (111); HRI-i (109); Control (32); Wnt5a (42); Wnt5a+7-NI (111) number of cells (*p  0.05, ***p = 0.00009). (**b**) Averaged sample traces of NMDAR-EPSCs obtained from the soma of CA1 pyramidal cells voltage-clamped at + 40 mV. Top panel, control currents at 30 and 60 min to show the stability of recordings; second, the current after Wnt5a bath application (1 h) and the selective GluN2B antagonist Ro25-6981 (3 µM); third panel, current after preincubation with HRI-i (1 µM) and Wnt5a addition; fourth, current after preincubation with CHX (50 µM) and Wnt5a. (**c**) The dot plots show the NMDA current amplitudes measured during every treatment relative to the control current in the cell before Wnt5a or drug application. The relative values for each condition were averaged and plotted as shown (Control, Wnt5a: N = 9 cells from 5 mice; Wnt5a + Ro: N = 10 cells from 5 mice; Ro: 6 cells from 5 mice; Wnt5a + HRI-i: N = 5 cells from 5 mice; HRI-i: N = 3 cells from 3 mice; Wnt5a+CHX: N = 3 from 3 mice; CHX: N = 3 from 3 mice). Lines represent the mean ± SEM; (*p  0.05, **p < 0.001, ***p = 0.00009). (**d**,**e**) Spontaneous action potentials (APs) in current clamp configuration. The presence of Wnt5a reduces the firing threshold for AP generation (**d**), and at the same resting membrane potential, Wnt5a induces more APs and also the generation of regular periods of bursts of activity (**e**). Notably, the presence of Ro prevented this feature. (**f**) The recordings obtained in (**b**) were normalized to obtain decay kinetics (tau, ms) in every tested condition. The dot plots show the relative values of the decay measurements and the mean ± SEM (*p < 0.05; **: *p* < 0.001).

We evaluated whether newly formed GluN2B subunits can produce functional NMDARs. Physiologically, NMDARs are activated through depolarization mediated by AMPA receptors and by the binding of glutamate and glycine or D-serine. To specifically activate NMDAR, we used its chemical ligand NMDA and its co-agonist glycine. Hippocampal neurons were pretreated for 1 h with control or Wnt5a medium alone or in the presence of HRI-i, 7-NI (Fig. 6a) or the endogenous chelator of Wnt5a ligand sFRP2 (Supplementary Fig. S7a). Neurons were incubated with the ratiometric probe Fura-2 AM and stimulated with NMDA plus glycine. The ratiometric probe is an accurate method of measuring calcium fluctuations while avoiding the false-positive signal coming from the unbound calcium (corresponding to 380 nm excitation). Our results showed that Wnt5a treatment increased the calcium signal by ~ 50–90% compared with untreated neurons or neurons co-incubated with HRI-i, 7-NI (Fig. 6a) or sFRP2 (Supplementary Fig. S7a) after NMDA stimulation. These data suggest that Wnt5a increases the production of functional channels through HRI activation and NO production. To determine the effect of Wnt5a on the NMDA currents, we performed whole-cell voltage clamp recordings from the soma of CA1 mouse hippocampal neurons at + 40 mV. We stimulated with an electrode in Schaffer collaterals in the presence of CNQX (an AMPA/kainate antagonist) to block the non-NMDA currents. We recorded the NMDA currents up to 60 min, showing no differences *vs* time 0 (Fig. 6b first panel). We determined the amplitude and decay kinetics of NMDA currents before and after the perfusion of Wnt5a for 1 h. The presence of Wnt5a caused a 1.5-fold increase in the NMDA current amplitude (control: 84.4 ± 11.2 pA *vs* Wnt5a: 117.1 ± 12.3 pA). The effect of Wnt5a was plotted relative to every cell baseline current level; the relative values of n = 9 cells were averaged and plotted (Fig. 6b first and second panel, c). Wnt5a also increased the decay time of the NMDA current (control: 139.9 ± 8.5 ms; Wnt5a: 240.3 ± 29.1 ms), indicating that channels stayed open for a longer time (Fig. 6b,c,f). To determine the contribution of GluN2B subunits to this current, we used the specific inhibitor of GluN2B subunits Ro 25–6981 maleate (Ro). The NMDA current decreased after 30 min of Ro addition and the reduction was even more evident after 60 min, demonstrating the role of GluN2B in basal NMDA currents. When Wnt5a was present in the recording media, we found that the presence of Ro reduced the amplitude of Wnt5a-induced NMDA currents (Wnt5a: 117.1 ± 12.3 pA *vs* Wnt5a + Ro: 31.1 ± 8.6 pA) (Fig. 6b second and third panels, c), demonstrating that a substantial part of the Wnt5a effect is mediated by GluN2B subunits. Interestingly, in the presence of Ro, the decay time of the current became faster, and the co-incubation with Wnt5a was unable to revert this effect (Wnt5a: 240.3 ± 29.1 ms; Wnt5a + Ro: 118.8 ± 22.4 ms), strongly suggesting that the increased decay time observed with Wnt5a is mainly due to the slow component induced by GluN2B (Fig. 6f).

To determine whether this effect is mediated by HRI activity and its regulation of GluN2B translation, we quantified NMDA currents in the presence of HRI-i or CHX in the recording solution (Fig. 6b fourth and fifth panels, c, f). To do this, cells were preincubated for 30 min with either HRI-i or CHX while recording the currents, and then, Wnt5a was added for 1 h. The inhibitors were added before Wnt5a stimulation to prevent the GluN2B translation. Both HRI-i and CHX prevented the effect of Wnt5a on the NMDA current amplitude (Wnt5a: 117.1 ± 12.3 pA *vs* HRI-i + Wnt5a: 83.5 ± 7.6 pA; CHX + Wnt5a: 64.4 ± 8.1 pA), and on the decay slope (Wnt5a: 240.3 ± 29.06 ms *vs* HRI-i + Wnt5a: 179.1 ± 17.5 ms; CHX + Wnt5a: 114.3 ± 20.9 ms), suggesting that translation is essential for the basal status of NMDA currents (Fig. 6b,c,f). We performed current-clamp recordings in the same conditions as described above to evaluate the excitability of the cells in the presence of Wnt5a. Ro reduced the increase in excitability (number of action potentials) in response to the same depolarizing current pulse caused by Wnt5a (Fig. 6b insets and Figs. 6d,e). Moreover, pre-incubation with HRI-i or CHX prevented the increase in Wnt5a-evoked action potentials (Fig. 6b, insets). From these results, we concluded that Wnt5a increases both, the number of active channels in the membrane and the open time of channels containing GluN2B subunits, and that a translational process mediated by HRI kinase triggers these effects.

### Wnt5a increased protrusions and synaptic contacts through HRI kinase activity in hippocampal neurons and synaptosomal preparations

**Figure 7:**
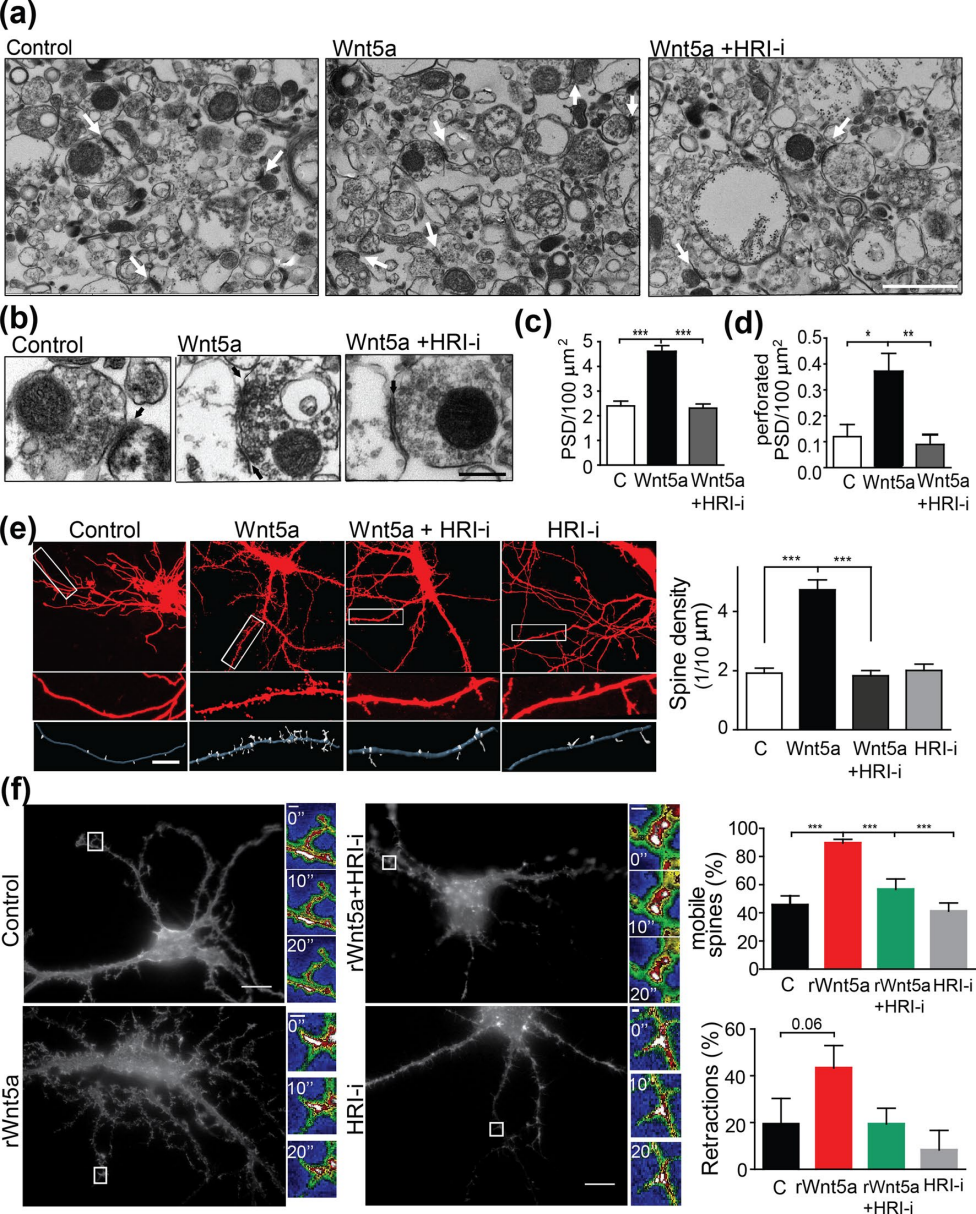
HRI kinase mediates Wnt5a-dependent increase in dendritic spine density. EM images of synaptosomal fractions from mouse brain. Representative images showing the number of PSDs (**a**,**c**) and the number of multiple synaptic contacts (**b**,**d**) after the treatment with control or Wnt5a conditioned medium or co-incubation withWnt5a and HRI-i. Quantifications of the synaptic contacts and number of multiple synaptic contacts per 100 µM were included. Scale bars: 1 μm and 200 nm, respectively. Bars show the mean ± SEM. N = 3 independent experiments. Control (50); Wnt5a (59); Wnt5a+ HRI-i (55) photos (*p  0.05, **p = 0.0008, ***p = 0.0001) (**e**) EGFP-transfected mouse hippocampal neurons (DIV10) and IMARIS 3D reconstruction. Representative images of cropped neurites from hippocampal neurons treated with control or Wnt5a conditioned medium, incubated in the presence or absence of the HRI inhibitor. Quantification of the number of spines per 10 μm of neurite. Bars show the mean ± SEM. N = 5 independent experiments. Control (82); Wnt5a (129); Wnt5a+ HRI-i (86); HRI-I (105) dendrites (***p = 0.0001). (**f**) EGFP-LifeAct-transfected hippocampal neurons in the absence (control) or presence of rWnt5a, rWnt5a+HRI-i or HRI-i alone for 1 h. Insets show the fluorescence intensity of one representative protrusion measured during the indicated time (shown in each inset, 0, 10, 20 s). The graphs show the percentage (%) of mobile protrusions and the % of retraction movement for each condition. N=3 independent experiments. Control (7); rWnt5a (12); rWnt5a+HRI-I (7); HRI-I (5), number of cells from 3 independent experiments. (*p  0.05, **p < 0.001, (***p = 0.0001).

Given that Wnt5a induces functional changes in NMDA currents and that synaptic function can regulate ultrastructural changes in synapses^27^, we hypothesized that Wnt5a might also mediate spine formation through GluN2B translation. To evaluate whether HRI kinase is involved in the Wnt5a remodeling of the PSD, we prepared synaptosomal fractions to show the local effect of HRI at the synaptic level. We observed an increase in the number of PSDs (Fig. 7a,c), and in the number of perforated PSDs after 1 h of Wnt5a exposure (Fig. 7b,d), which were completely blocked by co-incubation with the specific HRI-i (Fig. 7a–d).

To assess whether changes mediated by Wnt5a and HRI affect the density of dendritic protrusions, we performed the same experiments in cultured hippocampal neurons. Wnt5a induced a 2.5-fold increase in the number of dendritic protrusions (Fig. 7e), an effect that was completely prevented by co-incubation with HRI-i demonstrating that the effect of Wnt5a is mediated by HRI.

The actin cytoskeleton is essential to spine formation, and F-actin is the most common form of actin in spines^28–30^. Therefore, we evaluated the dynamics of F-acting during Wnt5a stimulation, transfecting hippocampal neurons with EGFP-LifeAct. After 1 h of rWnt5a treatment, we observed more spines (supporting our previous findings), which were more mobile than in non-treated (control) cells or cells treated with HRI-i alone or in combination with rWnt5a (Fig. 7f, 1, 2, 3 and 4). Detailed analysis of the actin flow and distribution demonstrates that spines previously treated with Wnt5a showed more actin flow but at a slower speed than non-treated, although the general distribution did not change [Supplementary Fig. S8, kymographs (b) and histogram (c)]. In particular, in neurons treated with rWnt5a the F-actin showed more retraction movements than protrusions, and these retraction movements tend to be higher compared to the control condition (see Fig. 7f for inset images of spines; see Supplementary Fig. S8 for an explanation of the analysis). We can speculate whether the protrusions produced after 1 h of rWnt5a treatment might be more filopodia (considering filopodia as protrusions with a neck larger than 2 µm), however we did not observe any difference in the % of filopodia in each condition (Supplementary Fig. S8f.).

## Methods

### Animals, statement of guidelines followed for the care and use of animal and ethical standards

Experiments were carried out on 2-month-old male C57BL/6J mice, unless otherwise specified. Mice were kept in a ventilated room, exposed to 12 h of light/dark cycle and room-controlled temperature (22 ± 3 °C) at the animal facility of the Pontificia Universidad Católica de Chile. Animals were fed with *ad libitum* standard commercial pellet diet and water. The authors state that the study was carried out in compliance with the ARRIVE and National Institutes of Health (NIH, Baltimore, MD, USA) guidelines. Experimental procedures under the number 160921007 (Ex CBB-122/2013) were approved by the Bioethical and Biosafety Committee of the Faculty of Biological Sciences of the Pontificia Universidad Católica de Chile. All the efforts were made to minimize animal suffering and reduce the number of animals used.

### Generation of control and Wnt ligand-containing conditioned media

Control and Wnt ligand-containing conditioned media were prepared from L-Cells (ATCC CRL-2648), and L-Wnt5a cells (ATTC CRL-2814) as previously described^16^.

### Primary culture of mouse hippocampal neurons

Mouse hippocampal neurons were isolated and cultured from C57BL/6J mice at embryonic day 18 as previously described^31^. Neurons were used for the experiments at day in vitro (DIV) 10.

### Transfection and spine image analysis

Transfection was performed by magnetofection, using NeuroMag (OZ Bioscience)^14^. First 60 × 10^3^ hippocampal neurons were seeded and transfected at DIV7 with 0.8 μg enhanced green fluorescent protein GFP plasmid (Clontech) to visualize the spines. At DIV10, hippocampal neurons were treated with control or Wnt5a conditioned medium plus 1 μM HRI inhibitor (HRI-i) (Janssen Research & Development). Confocal images at 1024×1024 resolution were acquired with an Olympus FluoView FV 1000 confocal microscope (Tokyo, Japan). Dendritic z- stacks were reconstructed into 3D images using the super-pass module of Imaris software (Bitplane USA, Oxford instruments, Concord MA 01742). Dendritic shafts and spines were traced using the filament mode. The protrusions below the 3 µm were considered for the 3D reconstruction. Accurate reconstruction of the diameters of the spine neck and head was carried out using the diameter function with a contrast threshold of 1.25.

### Electron microscopy from hippocampal slices (EM)

Hippocampal slices were prepared from brains of 2-month-old C57BL/6J mice as described previously^32^. Slices were incubated in cold artificial cerebrospinal fluid (ACSF) (126 mM NaCl, 3 mM KCl, 1.25 mM NaH_2_PO_4_, 25 mM NaHCO_3_, 10 mM glucose, 2 mM MgSO_4_, 2 mM CaCl_2_, 3 mM sodium pyruvate, 0.5 mM sodium ascorbate, pH 7.4) and kept oxygenated (95% O_2_ / 5% CO2) until treatments.

Mouse hippocampal slices were treated with control or Wnt5a conditioned medium (1/10 dilution; time of incubation is indicated in each figure) and used for EM analysis according to standard procedures^33^. The CA1 region of the hippocampus was selected for examination by EM from 1 mm sections stained with toluidine blue for light microscopy. Ultrathin sections were cut with a Reichert Ultramicrotome (Newark, DE), placed on 300-mesh copper EM grids, stained with uranyl acetate and lead citrate, and examined using a Phillips Tecnai 12 Transmission Electron Microscope (Philips Electron Optics, Holland) at 80 kV. Digital images obtained were analyzed by using ImageJ (NIH, USA) as previously described^34^.

### Synaptosomal preparation, treatments and image analysis

Synaptosomal fractions were obtained from 2-month-old male C57BL/6J mice. The procedure was carried out as previously reported^35^. Synaptosomes were treated with control or Wnt5a-containing conditioned medium or with Wnt5a-containing medium plus 1 μM HRI-i for 1 h (HRI-i: N-(2,6-dimethylbenzyl)-6,7-dimethoxy-2H-[1]benzofuro[3,2-c]pyrazol-3-amine hydrochloride^36^ (pretreated with HRI-i for 30 min). Synaptosomal solution was centrifuged at 4 °C at 24,000 *g* for 20 min. Pellets were cut into ultrathin sections (80 nm) and stained as described above. ImageJ software was used to identify PSD areas and to perform quantitative analyses.

### NO measurements by incubation with DAF-FM in hippocampal mouse neurons

The following experiments were performed as described by^37^ with some modifications. First, 30 × 10^4^ DIV10 hippocampal neurons were seeded on 25 mm coverslips and incubated with 2.5 µM DAF in a solution containing the following (in mM): 132 NaCl, 4.2 KCl, 1.8 CaCl_2_, 5 D-Glucose, 10 HEPES pH 7.4, for 15 min at room temperature. Then, the coverslips were washed for 15 min with the solution previously described. The recording solution is supplemented with 500 µM L-Arg as a source of NO production. The variations in cytosolic NO are presented as ∆F/F_0_ of the emitted fluorescence after excitation at 488 nm relative to the ratio measured prior to stimulation (first minute before application of the stimuli).

### DNA transfection of HT22 cells and primary cultures to perform luciferase assay

HT22 cells were seeded at 15 × 10^3^ cells per well in 96-well plates. Afterwards, a total of 50 ng DNA/well (25 ng *Renilla* luciferase + 25 ng CMV-luciferase vectors; 25 ng *Renilla* luciferase + 25 ng GluN2B-5′UTR CMV-luciferase vectors; 25 ng *Renilla* luciferase + 25 ng GluN2B-5′UTR triple mutant CMV-luciferase constructs) was transfected using the JetPEI transfection reagent (PolyPlus, Korea). The medium was replaced after 4 h and treatments were carried out at 48 h to allow sufficient gene expression. For primary hippocampal cultures, 50 × 10^4^ cells per well were seeded in 6-well plates, and at DIV8 were transfected with a total of 4 µg of DNA per well with NeuroMag (described above). At DIV10 neurons were incubated with Wnt5a medium for 1 h, and then lysed to measure luciferase/*Renilla* activities following the manufacturer’s instructions for the Dual-Glo Luciferase Assay System (Promega). Luminescence was read using a luminescence reader (Turner Designs Luminometer Model TD20/20).

### HRI kinase activity assay

HRI activity and the inhibitory ability of the HRI-i was determined using a luminometric assay as previously described for other kinases inhibitors^38^. For this assay, 1 μM HRI-i diluted in kinase buffer (20 mM Tris pH 8, 50 mM KCl, 25 mM MgCl2 and 1 µM phenylmethysulfonyl fluoride) was added to a reaction mixture composed of 5 μM eIF2α peptide substrate (Santa Cruz Biotechnology), immunoprecipitated HRI from mouse cortex/hippocampus (goat, Santa Cruz Biotechnology) and 10 µM ATP, in a final volume of 50 μl. After 5 min of incubation at room temperature, the enzymatic reaction was stopped by adding 50 µL of Kinase Glo (Promega). After 10 min of stabilization, the luminescence was measured with a Turner Designs Luminometer model TD20/20. The luciferase signal was proportional to the amount of ATP, and the data are expressed as the inverse of the ATP amount to determine the fold kinase activity.

### Western blot experiments

First, 50 × 10^4^ primary neurons were seeded per well into 6-well plates, and treated at DIV10 for 1 h at 37 °C with control or Wnt5a-containing conditioned medium or with recombinant Wnt5a (rWnt5a, R&D systems) (300 ng/mL) or were pretreated with 1 µM HRI-i or 100 µg/mL CHX (Sigma Aldrich) for 30 min -and then incubated with conditioned medium or rWnt5a. Cells were lysed with RIPA buffer supplemented with phosphatases and proteases inhibitors. Then, 60 µg of protein from hippocampal neurons or synaptosomes was loaded into 8–10% acrylamide:bisacrylamide gels. Primary antibodies used were: GluN2B from NeuroMab (1:5), Abcam (1:700) and Alomone (1:350), actin (Abcam 1:10,000), PS51-eIF2α (Abcam 1:500) and eIF2α (Abcam 1:500). Secondary antibodies (Abcam) were used at a dilution of 1:5000.

### Immunogold staining

Hippocampal slices were fixed with 4% PFA for 4 h at 4 °C and incubated with phosphate buffer (0.2 M Na_2_HPO_4_, 0.2 M KH_2_PO_4_, pH 7) overnight at 4 °C. Then, the CA1 region from the hippocampus was selected and placed into a grid. The antibodies used were a primary rabbit GluN2B antibody (1:700 Abcam) and a secondary 5 nm colloidal gold-conjugated antibody (Sigma). After, the grids were contrasted with 1% uranyl acetate for 5 min, photos were acquired at 43,000× with a Phillips Tecnai 12 Transmission Electron Microscope.

### Transfection of small interfering RNA and short hairpin RNA and viral infection

Small interfering RNAs (siRNAs) that were non-directed (siC) or were directed against HRI (siHRI) were obtained from QIAGEN (SI00105784, target sequence 5′-CTGATTAAGGGTGCAACTAAA-3′). Short hairpin RNAs (shRNAs) that were non-directed (shC) or were directed against HRI (shHRI, combination of 2 different shRNAs) cloned into a pLKO-tGFP vector were purchased from SIGMA (CSTDNA, SHCLND, CTGATTAAGAGCGCAACTAAA, ACAAACGTCACGCTACTTAAA). Transfections with 250 nM of siC or siHRI in 50 × 10^4^ neurons (WB) or 65 × 10^3^ (cotransfected with eGFP for the IF) at DIV8 and 0.8 ug of shC or shHRI in 65 × 10^3^ neurons at DIV6-7 respectively were performed by magnetofection as described above.

Third-generation lentivirus particles expressing shRNAs targeting HRI kinase and control shRNAs were prepared. Briefly, HEK293T cells were plated at 5 × 10^6^ cells per 100 mm plate for a total of three plates. After 24 h, cells were co-transfected with 14.4 μg of shRNA-expressing lentiviral vectors, with 9.2 μg of packaging vector (pMDLg/pRRE), 3.6 μg (pRSV-Rev) and 4.9 μg (pMD2.G) of envelope vectors using 2.5 µM polyethylenimine MAX (#24765, Polysciences, Inc) pH 7.1. After 4 h, the medium was replaced by a fresh medium containing 1% FBS. Lentivirus-containing supernatant was harvested 72 h post-transfection, centrifuged to eliminate cell debris, filtered through 0.22 μm cellulose acetate filters, and concentrated by two rounds of centrifugation using AMICON filters of 100 kDa (UFC910024, Millipore) at 4500 g using a swing bucket rotor. Transduction of 50 × 10^4^ neurons per well in 6-well plates was performed at DIV3, and neurons were analyzed by quantitative RT-PCR (qRT-PCR).

### RNA extraction, retrotranscription and quantitative PCR.

RNA extraction was performed via the Trizol method from 50 × 10^4^ neuron per weel in 6-well plates, and ~ 2 µg was used for retrotranscription with SuperscriptIII (1843289 Invitrogen). Primers for the quantitative PCR for HRI (Fw:5′-AACCCGCTCCACTCCAAAC-3′; Rv: 5′-GAGTGATGGCTCTGTTGTGGT-3′) and GluN2B (Fw:5′-CATTTATCCTTCGTCTTTCTTATGTG-3′; Rv:5′-CAACACCAACCAGAACTTG-3′) were designed and obtained from IDT. The results were evaluated with the 2^-∆∆CT^ method and normalized to GAPDH levels.

### Immunofluorescence staining

Transfected neurons were treated at DIV10 with rWnt5a for 1 h and then washed thrice with PBS Ca^2+^/Mg^2+^ (100 µM/1 mM), fixed with a freshly prepared solution of 4% PFA-sucrose in PBS for 20 min and permeabilized for 5 min with 0.2% Triton X-100 in PBS. Neurons were incubated with primary antibody (GluN2B Abcam, 1:700) overnight at 4 °C in a wet chamber. Secondary antibody coupled to 555 nm fluorophore was applied at a 1:500 dilution for 30 min at 37 °C.

### Measurements of intracellular Ca^***2+***^ in mouse hippocampal neurons

Intracellular Ca^2+^ measurements were carried out in DIV10 hippocampal neurons loaded with the ratiometric probe Fura-2 AM at 4.5 µM for 30 min. Before the assay, neurons were treated for 1 h with control or Wnt5a medium or with Wnt5a plus 1 µM HRI-i (JANSEN), 10 µM 7NI (SIGMA) or 250 nM sFRP2 (R&D systems), with a pretreatment of 30 min for all of these inhibitors. The experiments were performed with an isotonic solution containing (in mM): 2.5 CaCl_2_, 132 NaCl, 4 KCl, 10 NaHCO_3_, 6 glucose, and 10 HEPES (305 mOsm/liter, pH7.4). Cytosolic Ca^2+^ levels were continuously recorded (each 5 s) for 1 min before (basal levels) and 6 min after application of 100 µM NMDA + 100 µM glycine. Variations in cytosolic Ca^2+^ are presented as the normalized ratio of the emitted fluorescence at 510 nm after excitation at 340 and 380 nm relative to the ratio measured prior to stimulation (first minute before application of the stimuli), and the area under the curve (AUC) after the addition of NMDA and glycine was integrated. Live Ca^2+^ imaging was performed with an Olympus spinning disc IX 81 microscope.

### Preparation of mouse brain slices and electrophysiological recordings

Acute coronal slices were prepared from wild-type (C57/BL6J) male and female mice at P15–P20. The procedures were previously described in detail^39^. Briefly, immediately after removing the brain it was placed in a beaker containing sucrose-based solution (at 4 °C) composed by (in mM): 85 NaCl, 75 sucrose, 3 KCl, 1.25 NaH_2_PO_4_, 25 NaHCO_3_, 10 dextrose, 3.5 MgSO_4_, 0.5 CaCl_2_, 3 sodium pyruvate, 0.5 sodium L-ascorbate and 3 myo-inositol (305 mOsm, pH 7.4). Coronal slices were cut at 350 µm, and transferred to a beaker containing the same solution but at 36 °C. Thirty minutes later, the solution was changed to recording solution (ACSF), composed by (in mM): 126 NaCl, 3.5 KCl, 1.25 NaH_2_PO_4_, 25 NaHCO_3_, 10 dextrose, 1 MgSO_4_, 2 CaCl_2_, 3 sodium pyruvate, 0.5 sodium L-ascorbate and 3 myo-inositol (305 mOsm), pH 7.4). Both the cutting solution and recording solution were thoroughly oxygenated with 95% O_2_/5% CO_2_ during all the procedures. The slices were kept at room temperature (22 °C) until use. At the time of recording, the slices were placed in a submerged-style chamber solution with ACSF at 30–32 °C perfused at a rate of 3–4 ml/min, under an upright infrared-differential interference contrast (IR-DIC) fluorescence microscope (Eclipse FNI, Nikon) equipped with a 40× water objective and a light-sensitive camera (TOPICA CCD Camera). *Voltage clamp recordings* We performed voltage-clamp whole cell recordings in the soma of CA1 pyramidal cells to record NMDA-evoked excitatory postsynaptic currents (EPSCs). To do that, we used a borosilicate glass electrode (World Precision Instruments, Sarasota, FL, USA) pulled on a P-97 Flaming/Brown Micropipette Puller (Sutter Instruments, Novato, CA, USA). The glass pipette, ranging from 3.5 to 4.2 MΩ, was filled with intracellular solution containing the following (in mM): 130 Cs-gluconate, 3.5 CsCl, 4 ATP-Mg, 0.3 GTP-Na, 10 Na-phosphocreatine, 1 EGTA, 3.5 QX-314- Cl, 10 HEPES and 0.4% Biocytin (286 mOsm, pH 7.4 adjusted with CsOH). Cesium-gluconate and QX-314-Cl are voltage-dependent potassium and sodium currents blockers, respectively. After the formation of a seal and successful transition to whole-cell configuration, series resistance was monitored and compensated between 75 and 80% (Table 2). Cells were voltage clamped at − 60 mV and + 40 mV and the evoked EPSC was recorded every 15 s by stimulating the Schaffer collaterals at a distance of 100–150 µm of the recording pipette with a bipolar concentric electrode (World Precision Instruments, Sarasota, FL, USA) connected to a stimulus generator ISO-Flex (A.M.P.I., Jerusalem, Israel). The recordings were performed in ACSF supplemented with CNQX (5 µM) and PTX (25 µM), to block AMPA and GABA receptors, respectively. The peak of the NMDA current amplitude was measured at +40 mV, and the decay tau (ms) was obtained using a single exponential fit before and after Ro 25-6981 maleate (Ro), a specific inhibitor of GluN2B subunit, and was fitted from the peak down to the steady state of the current. To determine the I–V of NMDA activated current, we applied successive depolarizing pulses, from − 60 mV to + 40 mV, every 10 mV. After we collected the current for control, the same cell was then perfused with Wnt5a, and several depolarizing voltages were applied to monitor the changes in current during 1 h. At the end, Ro was applied to evaluate the GluN2B component; the cell was monitored, and the changes in current were compared with the cell’s own control values. It is noteworthy that the internal solution was freshly made for these experiments and we performed several experiments control just to check the stability of our recordings during an hour of patch recordings. In the experiments with HRI-i and CHX, these drugs were applied 30 min before Wnt5a and co-incubated for another 1 h. Recordings with a stable access resistance (R_a_) through the experiment were retained for additional analysis; in our hand, series resistance varies from 7 to 17 MΩ. We applied a small voltage step (-5 mV, 100 ms) through the experiments and those that change more than 20% were discarded for analysis. At least 10 cells from each condition were used for analysis. *Current clamp whole-cell recordings* For current-clamp recordings, the intracellular solution contains (in mM): 130 K-gluconate, 7 KCl, 10 HEPES, 4 ATP-Mg, 0.3 GTP-Na, 10 Na-phosphocreatine and 0.4% Biocytin (286 mOsm, pH 7.4 adjusted with KOH). Steps of positive current and negative current were injected to the soma to examine the intrinsic membrane properties of the recorded neuron. Cells fulfilling the following criteria were considered acceptable for further analysis: stable membrane potential less than − 60 mV (with no injected current), overshooting spike, and a balanced series resistance < 20 MΩ compensated over 75%. Series resistance compensation and capacitance neutralization were monitored and compensated throughout the experiment using a built-in bridge circuitry balance. Membrane potential was tracked throughout the whole experiment in the absence of a holding current. Just to examine the excitability properties of the cell at various membrane potentials, a direct current was applied through the recording electrode. Only recordings with a stable input resistance (R_in_) along the experiment were retained for additional analysis. The input resistance (R_in_) was systematically measured along the experiment (Table 2). It was calculated as the slope of the linear fit of the voltage-current measurements of the response of the resting membrane potential to current injection steps of amplitude − 50 pA to + 50 pA in 10 pA increments and 700 ms duration. Additionally, we controlled that the voltage sag ratio, calculated as the peak voltage deflection divided by the amplitude of the steady-state voltage deflection, in response to hyperpolarizing DC pulses of 700 ms were stable during long recordings (Supplementary Fig. S7b). The signals for both voltage-clamp and current-clamp were acquired using a MultiClamp 700B amplifier (Axon CNS, Molecular Devices LLC), low-pass filtered at 10 kHz, digitally sampled at 30 kHz and recorded through a Digidata-1440A interface (Axon CNS, Molecular Devices) and PClamp 10.3 software. Values are written as mean ± SEM, and the statistical comparisons were made with ANOVA.

**Table 2:** Electrophysiological parameters measured during prolonged experiments.

	Control	30 min	60 min
Resting membrane potential (RMP, mV)	− 65.5 ± 0.96		
Input resistance (R_in_, MΩ)	165.9 ± 5.7	156.9 ± 5.3	154.3 ± 7.7
Series resistance (R_s_, MΩ)	10.8 ± 3.1	12.3 ± 2.8	13.6 ± 4.8
Capacitance (pF)	92 ± 12		
Sag amplitude (mV)	5.5 ± 0.3	4.5 ± 0.7	3.5 ± 0.8
Number of action potentials (AP, to 100 pA)	16 ± 6	15 ± 4	14 ± 7
Action potential amplitude (from resting, mV)	96 ± 4	91 ± 3	87 ± 5

We measured different passive and active membrane parameters to determine that our recordings are feasible to be considered for further analysis. During voltage-clamp experiments, our R_s_ values range between 7 and 17 MΩ and were thoroughly monitored along the experiment. In current-clamp experiments we checked R_in_, sag amplitude, number and amplitude of AP (n = 10 cells, 5 animals).

### EGFP-LifeAct transfection and video analysis

LifeAct experiments were performed as described previously^40^ with some modifications. Briefly, 35 × 10^4^ hippocampal neurons in a 6-well plate were magnetofected with 2 µg of EGFP-LifeAct at DIV7. At DIV10 neurons were untreated or treated for 1 h with rWnt5a, rWnt5a+HRI-i or HRI-i. Then, EGFP-LifeAct fluorescence was recorded for 5 min in a 37 °C chamber with a bath solution containing (in nM): 25 HEPES, 119 NaCl, 2.5 KCl, 2 CaCl_2_, 2 MgCl_2_ and 30 glucose, at pH 7.4. Photos were obtained with a 100 × immersion oil objective at a resolution of 1344 × 1024 in an Olympus spinning disc IX 81 microscope. Actin flow and retraction analysis were performed in a semi-automated way using Image J software. At least 5 regions of interest (ROI) from dendrites from each neuron were used from each video. Using the KymoResliceWide plugin, we obtained the kymographs. For each protrusion in the kymograph, we established a line as a ROI and obtained the angle (*α*) of the vertical line representing the original position over time. The sine of *α* could then be used to solve the distance over which the protrusion extends or retracts. The time was solved using the scale µm/pixel from each kymograph. In this way, we could calculate the actin flow (µm/min). The mobile protrusions were those showing a deviation (*α*) from the vertical line representing the same position over time. The immobile had *α=*0 (Supplementary Fig. S8 a,b)*.* The % of mobile protrusions was calculated as the % of mobile protrusions/total number of protrusions. The retraction and protrusion percentage were analyzed by drawing from 2 to 3 squares per protrusion (Supplementary Fig. S8e). The displacement of maximum fluorescence through the squares allows us to measure the protrusion/retraction movements (Supplementary Fig. S8d). The retraction was considered when the peak of mean intensity fluorescence moved from the square head (purple square) to the squares middle (light grey) or initial neck (dark grey). Protrusions were considered when the peak of mean intensity fluorescence moved from the square initial neck to the squares middle neck or head. The % of retraction was calculated as % of retraction movements/ total number of mobile protrusions. Ten protrusions per photo were analyzed.

### MTT viability assay

For this assay, 10^5^ primary neurons/well were seeded in a 96-well plate and at DIV10, they were treated for 1 h with conditioned medium (control or Wnt5a) or co-incubated with conditioned medium and HRI-i (applied 30 min before the conditioned medium). After treatment, cells were incubated with MTT (Sigma) at a final concentration of 1 mg/mL for 2 h. Then, the medium was removed, and 100 µL of DMSO was added. Absorbance was read at 550 nm. N = 6 independent experiments.

### Quantification and statistical analysis

Analysis, quantification and statistical analysis of the data were performed using the following softwares: ImageJ, GraphPad Prism 5.0, PClamp 10.3, Spike2 and OriginPro8. The data are shown as the mean ± standard error of the mean (SEM) from at least 3 independent experiments, unless indicated otherwise in the figure legends. The graphs with less than 5 independent experiments are depicted with dots. *p* values were obtained using one-way ANOVA, as well as the *post hoc* Bonferroni correction for multiple comparisons or Dunnett’s Multiple Comparison Test to compare a treatment with the control condition and Student’s t-test to compare two experimental conditions. Significant differences are indicated by asterisks as follows: *p<0.05; **p<0.001; ***p<0.0001.

## Discussion

We have previously reported that Wnt5a modulates synaptic function through a specific action at the postsynaptic terminal^12,41,42^ inducing PSD-95 clustering^8^ and spine morphogenesis in rat hippocampal neurons^10^. PSD-95 is the major scaffold protein in the postsynaptic terminal and is required to sustain the molecular organization of spines^43^. The accumulation of PSD-95 in dendritic spines is associated with increased spine lifetime^44^. Both observations indicate that PSD size and organization are directly related to spine stability^45^. To determine whether the changes observed in PSD-95 clustering in response to Wnt5a correlate with a dynamic modification of spine structure, we measured PSD size in mouse hippocampal slices. Structural analysis of dendritic spine remodeling during activity indicated that there is a positive correlation between spine head volume, PSD area, presynaptic active zone area and synaptic strength^46–49^. Consistent with this idea, mouse slices treated with Wnt5a showed a maximum increase in PSD length and area at 1 h. After 2 h of Wnt5a exposure, the PSD length was reduced but the number of synaptic contacts, and spines with segmented PSD increased. These results suggest that the Wnt5a-mediated reduction in the average PSD length is a consequence of the generation of new dendritic spines, as we show here and in a previous report^10^. In mouse hippocampal cultures we already observe changes in dendritic spine density at 1 h of Wnt5a treatment. We think that these data from different models reinforce our hypothesis about the role of Wnt5a in the differentiation of dendritic spines, although the time of exposition needed to observe changes will be different in culture *vs* slices.

Wnt5a-mediated effects also increased the formation of perforated PSDs in synaptosomal preparations, producing a structural remodeling of the spine. This effect might require first an increase in PSD size, then the generation of new dendritic spines and the morphological modifications of the spine associated with spine maturation and synaptic plasticity, i.e., the segmentation of the PSD^50–52^. The effect of Wnt5a in the maturation of the spines has already been shown, through the increase of PSD95 clustering, and the increase of the number of spines containing PSD95^8^. The formation of perforated PSDs in spines has also been associated with the restructuration of dendritic spines in response to LTP induction and synaptic potentiation^53–55^ and it depends on the activation of NMDA receptors^55^. Interestingly, the activation of the non-canonical Wnt signaling, through Wnt5a, has been shown to enhance hippocampal LTP and to modulate synaptic plasticity^11,32^. Remarkably, in a previous work we had suggested that the effect of Wnt5a was to incorporate ‘new cells’, that were previously silent, into a functional oscillating neuronal circuit^39^. These data suggest that perforated spines are the substrate of those physiological changes and that one hour of Wnt5a exposure is enough to induce substantial circuital effects. Our results support these findings, and strongly suggest that Wnt5a participates in dendritic spine remodeling to enhance synaptic function and strength.

Our previous studies demonstrated that Wnt5a induces NO production^17^, associated with an increase in cytosolic calcium, which is required for the nNOS enzymatic activity in glutamatergic neurons^56,57^. Among other effects, NO has an important role in LTP and memory formation^58^ and interacts with the heme group of HRI kinase, allowing the exposure of HRI’s catalytic site and subsequent activation^25,59^. HRI kinase phosphorylates eIF2α^60^, inhibiting the translation of most proteins^61^, but allowing for the translation of mRNAs with more than one ORF in their 5′UTRs^62^. Excessive p-eIF2α has been associated with cognitive impairment in Alzheimer’s disease^63^, but it also plays physiological roles related to the formation of new, growing dendritic spines. It has been reported that under physiological conditions, the phosphorylation of eIF2α through HRI kinase activates BACE-1 translation and increases spine number^64^. Our results showed that the inhibition of HRI kinase prevented the Wnt5a-induced increase in spine density. Moreover, using EM on mouse synaptosomal fractions, we determined that HRI kinase is also responsible for the increase in postsynaptic contacts and perforated PSDs produced by Wnt5a.

HRI kinase has previously been reported to be involved in the regulation of GluN2B expression through the activity of NO^22^. The GluN2B subunit of NMDAR is one of the most important proteins in the forebrain and is present at high levels in PSDs in that region^65–67^. In the PSD, it binds directly to PSD-95 and allows the stabilization and coupling of NMDAR with downstream signaling molecules such as NOS^68^ and Kalirin-7, a guanine nucleotide exchange factor that regulates dendritic spines^69^. Thus, the regulation of GluN2B subunit expression by Wnt5a through HRI kinase could have direct effects on postsynaptic formation, remodeling and function. In particular, Akashi and colleagues presented compelling evidence that CA3-GluN2B knockout (KO) mice have a lower F-actin/G-actin ratio in the synaptosomal fraction, showing an effect on the actin cytoskeleton dynamics. F-actin is most concentrated in dendritic spines and its depolymerization dramatically decreases the numbers of synaptic clusters of NMDARs, AMPARs, CaMKIIα and PSD scaffolds. Because of this effect, CA3-GluN2B-KO mice also showed a 24% reduction in dendritic spine density^70^. Moreover, several studies have demonstrated that the synaptic function can restructure synaptic formation and plasticity^27,71^. Our results also show that Wnt5a increases calcium entry and NMDA currents by the activation of HRI kinase, probably modifying the neuronal transmission and thus synaptic function and structure. In addition, our live imaging experiments studying F-actin dynamics show that Wnt5a, through HRI, also increases the F-actin mobility, mainly producing retraction, probably to ensure spine maturation. Here, we found that GluN2B is a novel postsynaptic target protein of Wnt5a, whose translation is mediated by HRI kinase, and has a key role in the effects of Wnt5a on PSD structure.

The effect of Wnt5a KO on hippocampal dendritic maintenance has recently been described^72^. Wnt5a KO mice show impaired CA1 NMDAR-dependent LTP, a reduction in GluN1 but no differences in GluN2 levels compared to wild-type mice. The authors noted that an altered GluN2A/2B ratio would be expected because reduction in GluN1 levels normally implies a decrease in GluN2A/2B protein. Although this study appears to be contradictory with our data, we described an active role for Wnt5a increasing GluN2B levels by translational de-repression through NO production and HRI kinase activation. Instead, the complete absence of Wnt5a might not decrease GluN2B subunits because, NO itself or other inducers of NO production could compensate for the role of Wnt5a in GluN2B translation. On the other hand, McQuate and coworkers, using DIV14 mature neurons did not observe differences in GluN2B expression under Wnt5a treatment^73^. The difference with our results can be explained by the fact that all our experiments were carried out at DIV10, during the synaptogenic period, when synapses are recently formed and the spine structure is still being established. Therefore, there is more plasticity at DIV10 than at DIV14.

**Figure 8:**
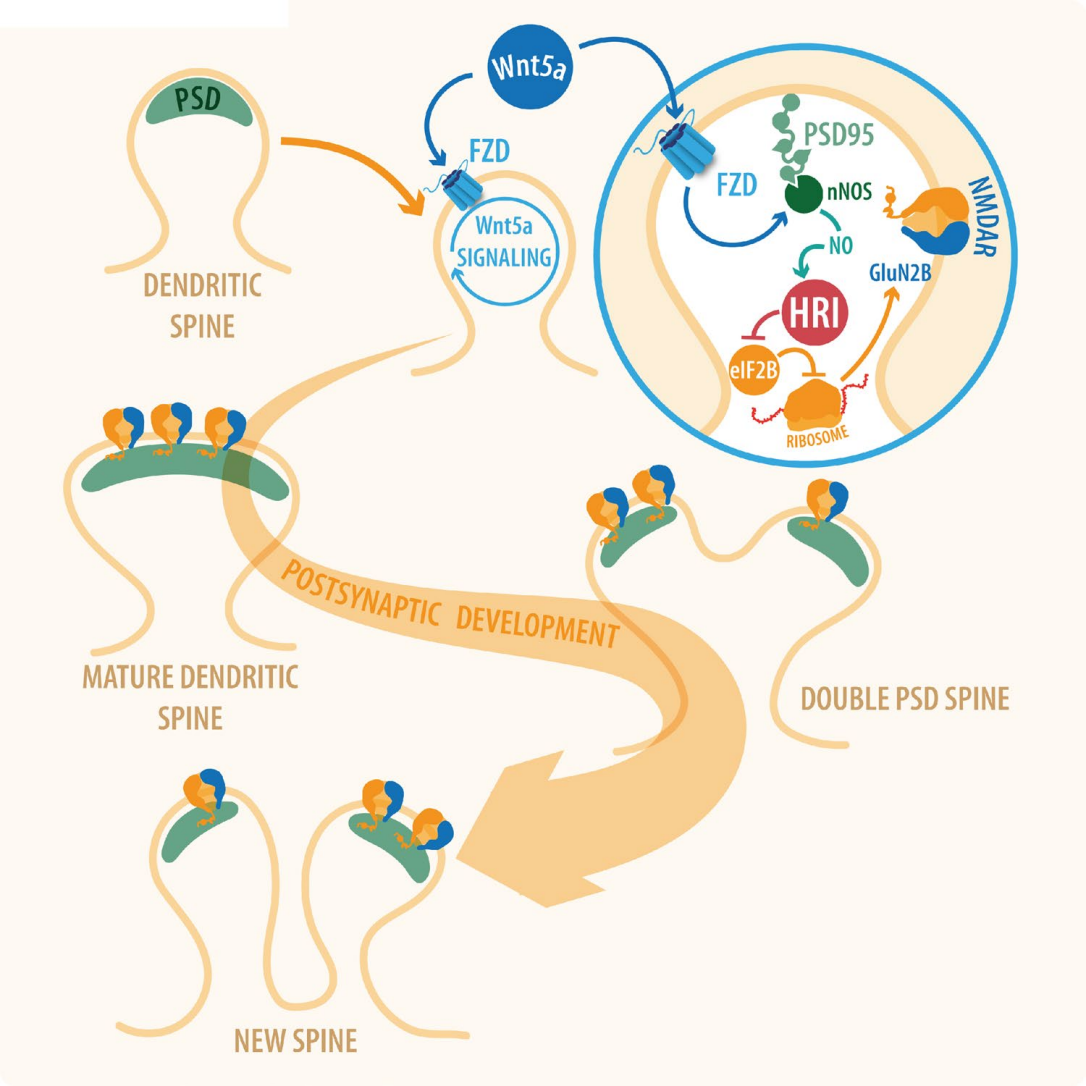
Wnt5a/NO/HRI signaling pathway promotes GluN2B translation and postsynaptic development. Wnt5a by binding to Fzd (Frizzled) receptor increases intracellular calcium and stimulates the production of NO, activating the HRI kinase. When HRI is active, it produces the translation of postsynaptic proteins like GluN2B subunits. This protein synthesis favors the maturation and formation of new dendritic spines. Felipe Serrano (Illustrative-science) designed the model of the signaling pathway.

In conclusion, our study demonstrates for the first time to our knowledge, that Wnt5a stimulates postsynaptic remodeling via a mechanism involving the activation of the Wnt5a-HRI kinase pathway and the translation of the GluN2B subunit. The structural changes would occur sequentially. First, there would be an enlargement of PSD structure, then its segmentation, and finally de novo formation of dendritic spines and new synaptic contacts (Fig. 8). Our results support the idea that the activation of non-canonical Wnt5a signaling plays a key role in postsynaptic development and the translation of important proteins for synaptic transmission.

## Supplementary Information

Supplementary Information.

Supplementary Video 1.

Supplementary Video 2.

Supplementary Video 3.

Supplementary Video 4.

## Abbreviations

CHX: Cycloheximide
HRI kinase: Heme-regulated eukaryotic initiation factor 2α kinase
KO: Knockout
LTP: Long term potentiation
NMDAR: N-methyl-D-aspartate receptor
NO: Nitric oxide
NOS: Nitric oxide synthase
PSD: Postsynaptic density
PSD-95: Postsynaptic density-95
uORF: Upstream open reading frame
5′UTR: 5′Untranslated region

## References

[1] Chen J, Park CS, Tang S-J (2006). Activity-dependent synaptic Wnt release regulates hippocampal long term potentiation. J Biol Chem.

[2] Dickins EM, Salinas PC (2013). Wnts in action: from synapse formation to synaptic maintenance. Front Cell Neurosci.

[3] Inestrosa NC, Varela-Nallar L (2014). Wnt signaling in the nervous system and in Alzheimer’s disease. J. Mol. Cell Biol..

[4] Nusse R (2012). Wnt signaling. Cold Spring Harb. Perspect. Biol..

[5] Shimogori T, VanSant J, Paik E, Grove EA (2004). Members of the Wnt, Fz, and Frp gene families expressed in postnatal mouse cerebral cortex. J. Comp. Neurol..

[6] Park M, Shen K (2012). WNTs in synapse formation and neuronal circuitry. EMBO J..

[7] Farías GG, Godoy JA, Cerpa W (2010). Wnt signaling modulates pre- and postsynaptic maturation: therapeutic considerations. Dev. Dyn..

[8] Farías GG, Alfaro IE, Cerpa W (2009). Wnt-5a/JNK signaling promotes the clustering of PSD-95 in hippocampal neurons. J. Biol. Chem..

[9] Cerpa W, Toledo EM, Varela-Nallar L, Inestrosa NC (2009). The role of Wnt signaling in neuroprotection. Drug News Perspect..

[10] Varela-Nallar L, Alfaro IE, Serrano FG (2010). Wingless-type family member 5A (Wnt-5a) stimulates synaptic differentiation and function of glutamatergic synapses. Proc. Natl. Acad. Sci. U. S. A..

[11] Cerpa W, Gambrill A, Inestrosa NC, Barria A (2011). Regulation of NMDA-receptor synaptic transmission by Wnt signaling. J. Neurosci..

[12] Inestrosa NC, Varela-Nallar L (2015). Wnt signalling in neuronal differentiation and development. Cell Tissue Res..

[13] Varela-Nallar L, Parodi J, Farías GG, Inestrosa NC (2012). Wnt-5a is a synaptogenic factor with neuroprotective properties against Aβ toxicity. Neurodegener. Dis..

[14] Ramírez VT, Ramos-Fernández E, Henríquez JP (2016). Wnt-5a/Frizzled9 receptor signaling through the Gαo-Gβγ complex regulates dendritic spine formation. J. Biol. Chem..

[15] Ortiz-Matamoros A, Arias C (2018). Chronic infusion of Wnt7a, Wnt5a and Dkk-1 in the adult hippocampus induces structural synaptic changes and modifies anxiety and memory performance. Brain Res. Bull..

[16] Cuitino L, Godoy JA, Farías GG (2010). Wnt-5a modulates recycling of functional GABAA receptors on hippocampal neurons. J. Neurosci..

[17] Muñoz FJ, Godoy JA, Cerpa W (2014). Wnt-5a increases NO and modulates NMDA receptor in rat hippocampal neurons. Biochem. Biophys. Res. Commun..

[18] Prast H, Philippu A (2001). Nitric oxide as modulator of neuronal function. Prog. Neurobiol..

[19] Guix FX, Uribesalgo I, Coma M, Muñoz FJ (2005). The physiology and pathophysiology of nitric oxide in the brain. Prog. Neurobiol..

[20] Prast H, Lamberti C, Fischer H (1996). Nitric oxide influences the release of histamine and glutamate in the rat hypothalamus. Naunyn Schmiedebergs Arch. Pharmacol..

[21] Ill-Raga G, Köhler C, Radiske A (2013). Consolidation of object recognition memory requires HRI kinase-dependent phosphorylation of eIF2α in the hippocampus. Hippocampus.

[22] Ramos-Fernández E, Tajes M, Ill-Raga G (2016). Glutamatergic stimulation induces GluN2B translation by the nitric oxide-Heme-Regulated eIF2α kinase in cortical neurons. Oncotarget.

[23] Ill-Raga G, Tajes M, Busquets-García A (2015). Physiological control of nitric oxide on neuronal BACE1 translation by heme-regulated Eif2-alpha kinase HRI induces synaptogenesis. Antioxid. Redox Signal..

[24] Purro SA, Dickins EM, Salinas PC (2012). The secreted Wnt antagonist Dickkopf-1 is required for amyloid β-mediated synaptic loss. J. Neurosci..

[25] Yun B-G, Matts JAB, Matts RL (2005). Interdomain interactions regulate the activation of the heme-regulated eIF 2 alpha kinase. Biochim. Biophys. Acta.

[26] Rosen MD, Woods CR, Goldberg SD (2009). Discovery of the first known small-molecule inhibitors of heme-regulated eukaryotic initiation factor 2?? (HRI) kinase. Bioorganic Med. Chem. Lett..

[27] Fukazawa Y, Saitoh Y, Ozawa F (2003). Hippocampal LTP Is accompanied by enhanced F-actin content within the dendritic spine that is essential for late LTP maintenance in vivo. Neuron.

[28] Capani F, Martone ME, Deerinck TJ, Ellisman MH (2001). Selective localization of high concentrations of F-actin in subpopulations of dendritic spines in rat central nervous system: a three-dimensional electron microscopic study. J. Comp. Neurol..

[29] Matus A, Ackermann M, Pehling G (1982). High actin concentrations in brain dendritic spines and postsynaptic densities. Proc. Natl. Acad. Sci..

[30] Honkura N, Matsuzaki M, Noguchi J (2008). The subspine organization of actin fibers regulates the structure and plasticity of dendritic spines. Neuron.

[31] Arrázola MS, Ramos-Fernández E, Cisternas P (2017). Wnt signaling prevents the Aβ oligomer-induced mitochondrial permeability transition pore opening preserving mitochondrial structure in hippocampal neurons. PLoS ONE.

[32] Vargas JY, Fuenzalida M, Inestrosa NC (2014). In vivo activation of Wnt signaling pathway enhances cognitive function of adult mice and reverses cognitive deficits in an Alzheimer’s disease model. J. Neurosci..

[33] Perkins EM, McCaffery JM (2007). Conventional and immunoelectron microscopy of mitochondria. Methods Mol. Biol..

[34] Arrázola MS, Inestrosa NC (2015) Monitoring mitochondrial membranes permeability in live neurons and mitochondrial swelling through electron microscopy analysis. In: Lossi L, Merighi A (eds) Neuronal Cell Death. Methods in Molecular Biology. Springer New York, pp 1254, 87–97 10.1007/978-1-4939-2152-2_7 25431059

[35] Myhre O, Fonnum F (2001). The effect of aliphatic, naphthenic, and aromatic hydrocarbons on production of reactive oxygen species and reactive nitrogen species in rat brain synaptosome fraction: the involvement of calcium, nitric oxide synthase, mitochondria, and phospholipase A. Biochem. Pharmacol..

[36] Rosen MD, Woods CR, Goldberg SD (2009). Discovery of the first known small-molecule inhibitors of heme-regulated eukaryotic initiation factor 2 alpha (HRI) kinase. Bioorganic Med. Chem. Lett..

[37] Molokanova E, Akhtar MW, Sanz-Blasco S (2014). Differential effects of synaptic and extrasynaptic NMDA receptors on a -induced nitric oxide production in cerebrocortical neurons. J. Neurosci..

[38] Baki A, Bielik A, Molnár L (2007). A high throughput luminescent assay for glycogen synthase kinase-3beta inhibitors. Assay Drug Dev. Technol..

[39] Oliva CA, Inestrosa NC (2015). A novel function for Wnt signaling modulating neuronal firing activity and the temporal structure of spontaneous oscillation in the entorhinal-hippocampal circuit. Exp. Neurol..

[40] Riedl J, Crevenna AH, Kessenbrock K (2008). Lifeact: a versatile marker to visualize F-actin. Nat. Methods.

[41] Inestrosa NC, Arenas E (2010). Emerging roles of Wnts in the adult nervous system. Nat. Rev. Neurosci..

[42] Rosso SB, Inestrosa NC (2013). WNT signaling in neuronal maturation and synaptogenesis. Front. Cell. Neurosci..

[43] Chen X, Nelson CD, Li X (2011). PSD-95 is required to sustain the molecular organization of the postsynaptic density. J. Neurosci..

[44] Cane M, Maco B, Knott G, Holtmaat A (2014). The relationship between PSD-95 clustering and spine stability in vivo. J. Neurosci..

[45] Ehrlich I, Klein M, Rumpel S, Malinow R (2007). PSD-95 is required for activity-driven synapse stabilization. Proc. Natl. Acad. Sci. U. S. A..

[46] Bosch M, Castro J, Saneyoshi T (2014). Structural and molecular remodeling of dendritic spine substructures during long-term potentiation. Neuron.

[47] Meyer D, Bonhoeffer T, Scheuss V (2014). Balance and stability of synaptic structures during synaptic plasticity. Neuron.

[48] Sorra KE, Harris KM (1998). Stability in synapse number and size at 2 hr after long-term potentiation in hippocampal area CA1. J. Neurosci..

[49] Straub C, Sabatini BL (2014). How to grow a synapse. Neuron.

[50] Calverley RK, Jones DG (1990). Contributions of dendritic spines and perforated synapses to synaptic plasticity. Brain Res. Rev..

[51] Geinisman Y, Morrell F, deToledo-Morrell L (1989). Perforated synapses on double-headed dendritic spines: a possible structural substrate of synaptic plasticity. Brain Res..

[52] Hering H, Sheng M (2001). Dendritic spines: structure, dynamics and regulation. Nat. Rev. Neurosci..

[53] Geinisman Y, De Toledo-Morrell L, Morrell F (1991). Induction of long-term potentiation is associated with an increase in the number of axospinous synapses with segmented postsynaptic densities. Brain Res..

[54] Geinisman Y, Detoledo-Morrell L, Morrell F (1996). Synapse restructuring associated with the maintenance phase of hippocampal long-term potentiation. J. Comp. Neurol..

[55] Neuhoff H, Roeper J, Schweizer M (1999). Activity-dependent formation of perforated synapses in cultured hippocampal neurons. Eur. J. Neurosci..

[56] Alderton WK, Cooper CE, Knowles RG (2001). Nitric oxide synthases: structure, function and inhibition. Biochem. J..

[57] Piazza M, Guillemette JG, Dieckmann T (2015). Chemical shift perturbations induced by residue specific mutations of CaM interacting with NOS peptides. Biomol. NMR Assign.

[58] Son H, Hawkins RD, Martin K (1996). Long-term potentiation is reduced in mice that are doubly mutant in endothelial and neuronal nitric oxide synthase. Cell.

[59] Igarashi J, Murase M, Iizuka A (2008). Elucidation of the heme binding site of heme-regulated eukaryotic initiation factor 2α kinase and the role of the regulatory motif in heme sensing by spectroscopic and catalytic studies of mutant proteins. J. Biol. Chem..

[60] de Haro C, Méndez R, Santoyo J (1996). The eIF-2alpha kinases and the control of protein synthesis. FASEB J..

[61] Clemens MJ (1994). Regulation of eukaryotic protein synthesis by protein kinases that phosphorylate initiation factor eIF-2. Mol. Biol. Rep..

[62] O’Connor T, Sadleir KR, Maus E (2008). Phosphorylation of the translation initiation factor eIF2α increases BACE1 levels and promotes amyloidogenesis. Neuron.

[63] Ma T, Trinh MA, Wexler AJ (2013). Suppression of eIF2α kinases alleviates Alzheimer’s disease-related plasticity and memory deficits. Nat. Neurosci..

[64] Ill-Raga G, Tajes M, Busquets-García A (2015). Physiological control of nitric oxide in neuronal BACE1 translation by heme-regulated eIF2α kinase HRI induces synaptogenesis. Antioxid. Redox Signal..

[65] Kim HG, Fox K, Connors BW (1995). Properties of excitatory synaptic events in neurons of primary somatosensory cortex of neonatal rats. Cereb. Cortex.

[66] Kornau HC, Schenker LT, Kennedy MB, Seeburg PH (1995). Domain interaction between NMDA receptor subunits and the postsynaptic density protein PSD-95. Science.

[67] Niethammer M, Kim E, Sheng M (1996). Interaction between the C terminus of NMDA receptor subunits and multiple members of the PSD-95 family of membrane-associated guanylate kinases. J. Neurosci..

[68] Aarts M, Liu Y, Liu L (2002). Treatment of ischemic brain damage by perturbing NMDA receptor- PSD-95 protein interactions. Science.

[69] Penzes P, Jones KA (2008). Dendritic spine dynamics–a key role for kalirin-7. Trends Neurosci..

[70] Akashi K, Kakizaki T, Kamiya H (2009). NMDA receptor GluN2B (GluR epsilon 2/NR2B) subunit is crucial for channel function, postsynaptic macromolecular organization, and actin cytoskeleton at hippocampal CA3 synapses. J. Neurosci..

[71] Bourne JN, Harris KM (2008). Balancing structure and function at hippocampal dendritic spines. Annu. Rev. Neurosci..

[72] Chen C-M, Orefice LL, Chiu S-L (2017). Wnt5a is essential for hippocampal dendritic maintenance and spatial learning and memory in adult mice. Proc. Natl. Acad. Sci..

[73] McQuate A, Latorre-Esteves E, Barria A (2017). A Wnt/calcium signaling cascade regulates neuronal excitability and trafficking of NMDARs. Cell Rep..

