# Supplementary material for: Wnt5a promotes hippocampal postsynaptic development and GluN2B-induced expression via the eIF2α HRI kinase
Source: Sci Rep. 2021 Apr 1;11:7395. doi: 10.1038/s41598-021-86708-y (PMC8016897; doi:10.1038/s41598-021-86708-y)
Supplement: Supplementary file 1 — Supplementary Information. [file 41598_2021_86708_MOESM1_ESM.pdf]

Supplementary information

Title page

Wnt5a promotes hippocampal postsynaptic development and GluN2B-induced expression via the eIF2α HRI kinase

Eva Ramos-Fernández[1,4♯], Macarena S. Arrázola[1,5*], Carolina A. Oliva[1*], Sebastián B. Arredondo[2], Lorena Varela-Nallar[2] and Nibaldo C. Inestrosa[1,3♯]

[1]Centro de Envejecimiento y Regeneración (CARE UC), Departamento de Biología Celular y Molecular, Facultad de Ciencias Biológicas, Pontificia Universidad Católica de Chile, Santiago, Chile, 8331150. [2] Instituto de Ciencias Biomédicas, Facultad de Medicina y Facultad de Ciencias de la Vida, Universidad Andrés Bello. [3]Centro de Excelencia en Biomedicina de Magallanes (CEBIMA), Universidad de Magallanes, Punta Arenas, Chile.[4] Current address: École polytechnique fédérale de Lausanne. [5] Current address: Centro de Biología Integrativa, Universidad Mayor

* Equal contribution

[♯]Corresponding author: Dr. Eva Ramos Fernandez at CARE UC Biomedical Center, Pontificia Universidad Católica de Chile, Av. Alameda 340, Santiago, Chile, postal code 8331150; e-mail: eva.ramosfernandez@epfl.ch

[♯]Corresponding author: Dr. Nibaldo C. Inestrosa at CARE UC Biomedical Center, Pontificia Universidad Católica de Chile, Av. Alameda 340, Santiago, Chile, postal code 8331150; e-mail: ninestrosa@epfl.ch

Abbreviated title: Wnt5a modulates PSDs through HRI kinase

Figure S1

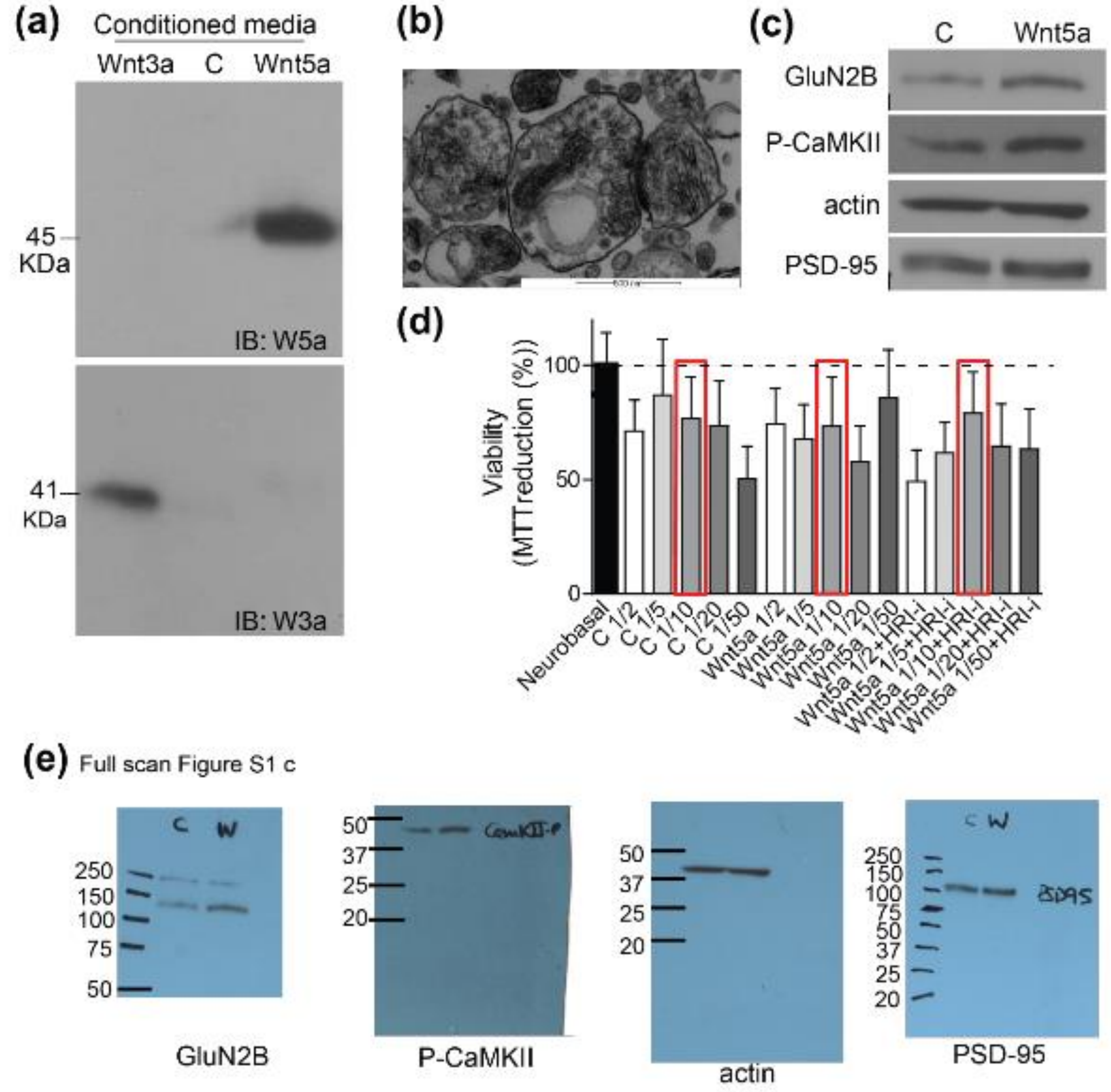

Figure S1. Reliability of conditioned media and synaptosomes *a) Representative western blot of control, Wnt5a-containing and Wnt3a-containing conditioned media. b) Representative transmission EM image of synaptosome isolation. c) Representative western blot of hippocampal neurons treated with Wnt5a and the signaling activation (p-CaMKII). d) Viability assay (MTT reduction %) of hippocampal neurons treated with conditioned media and HRI-i. N=6 independent experiments. e) Full-length blots of Figure S1c.*

Figure S2

(a)

(b)

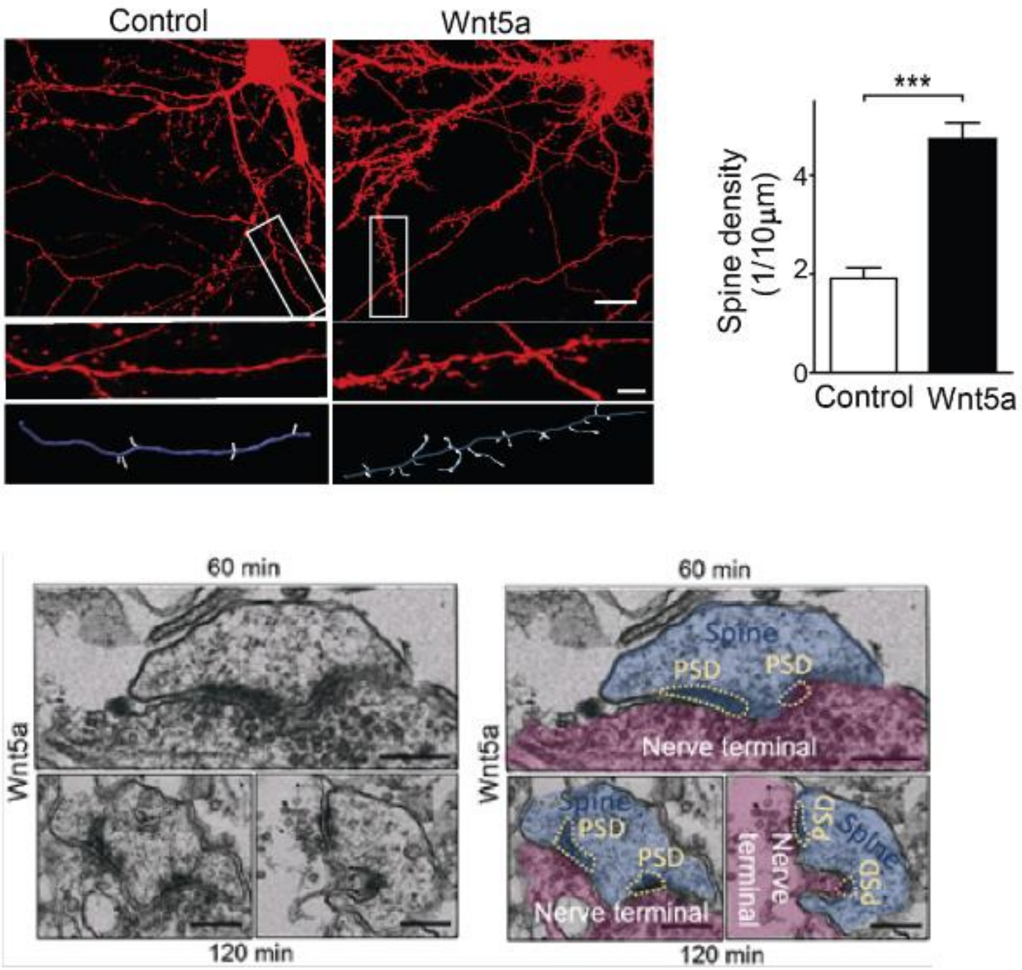

Figure S2. Wnt5a increases spine density in DIV10 neurons. *Representative images of 3D reconstructed dendrites and spines (left panel) from neurons transfected with EGFP (right panel, neurites shown in red) at DIV7 and treated at DIV10 with control and Wnt5a conditioned media. Scale bar: 3 μm. N=5 independent experiments; Control (82); Wnt5a (129) dendrites. ***: p= 0.00009 b) Colored EM images in Figure 2a showing Postsynaptic ending (Spine, in blue), Nerve terminal (pink) and PSD region (yellow).*

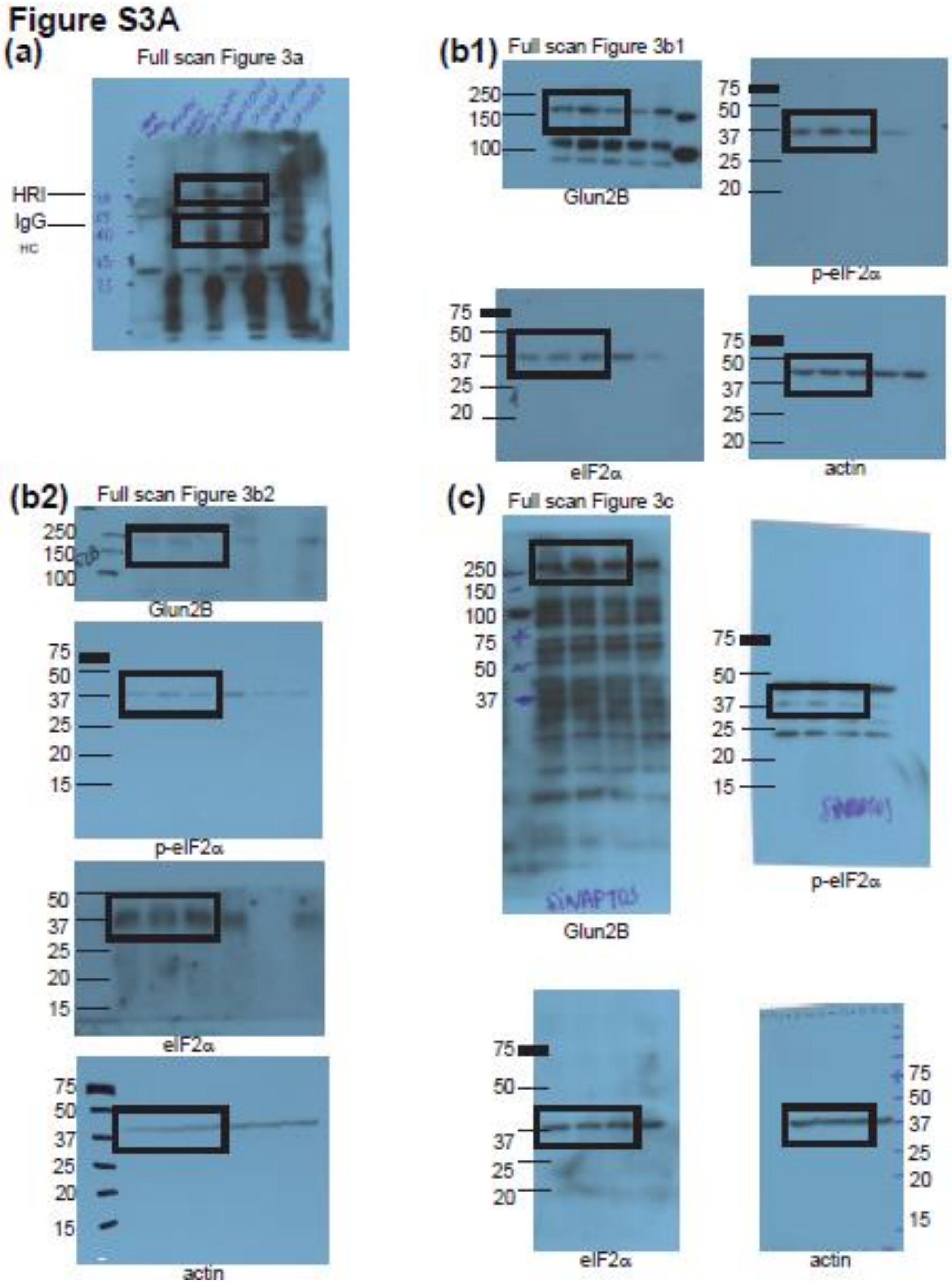

Figure S3A. Full-length gels of Figure 3

Figure S3B

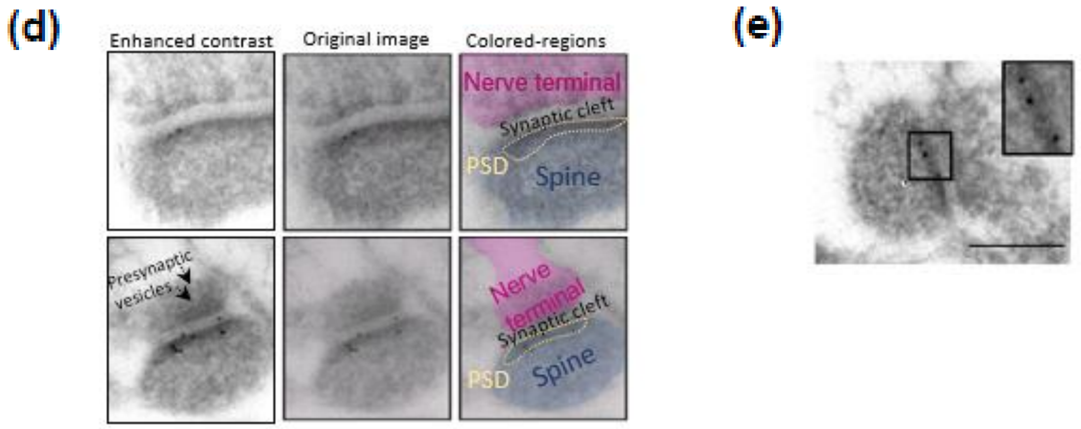

Figure S3B. Immunogold images. *(d) EM images of Figure 3 d with enhanced contrast and colored regions. Nerve terminal (pink), synaptic cleft, presynaptic vesicles, Spine (blue) and PSD region (yellow) are highlighted. (e) Immunogold image showing PSD-95 (larger diameter gold particle, in the middle) and GluN2B (two gold particles at the extremes of PSD).*

Figure S4

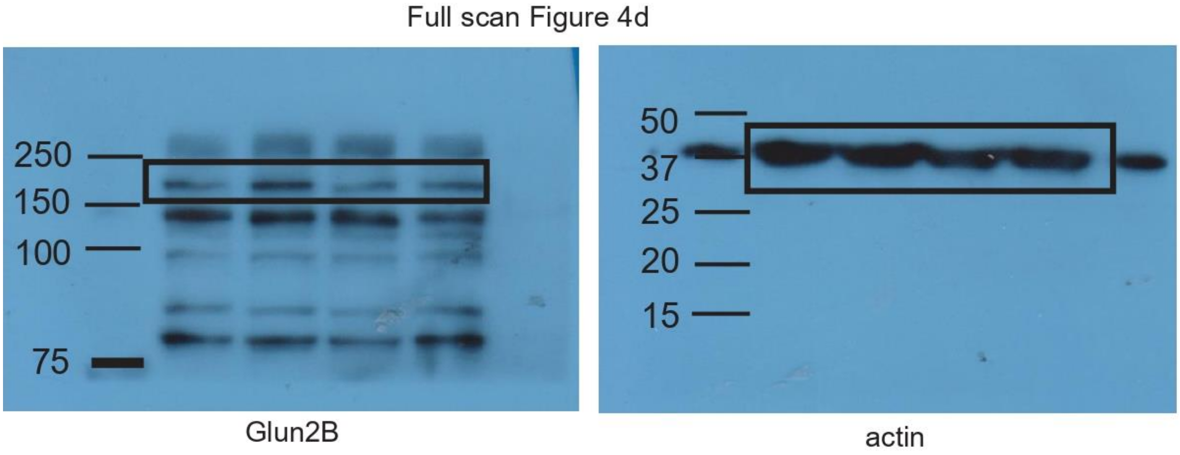

Figure S4. Full-length blots of Figure 4. The protein ladder was loaded into the extreme wells of the blot.

Figure S5

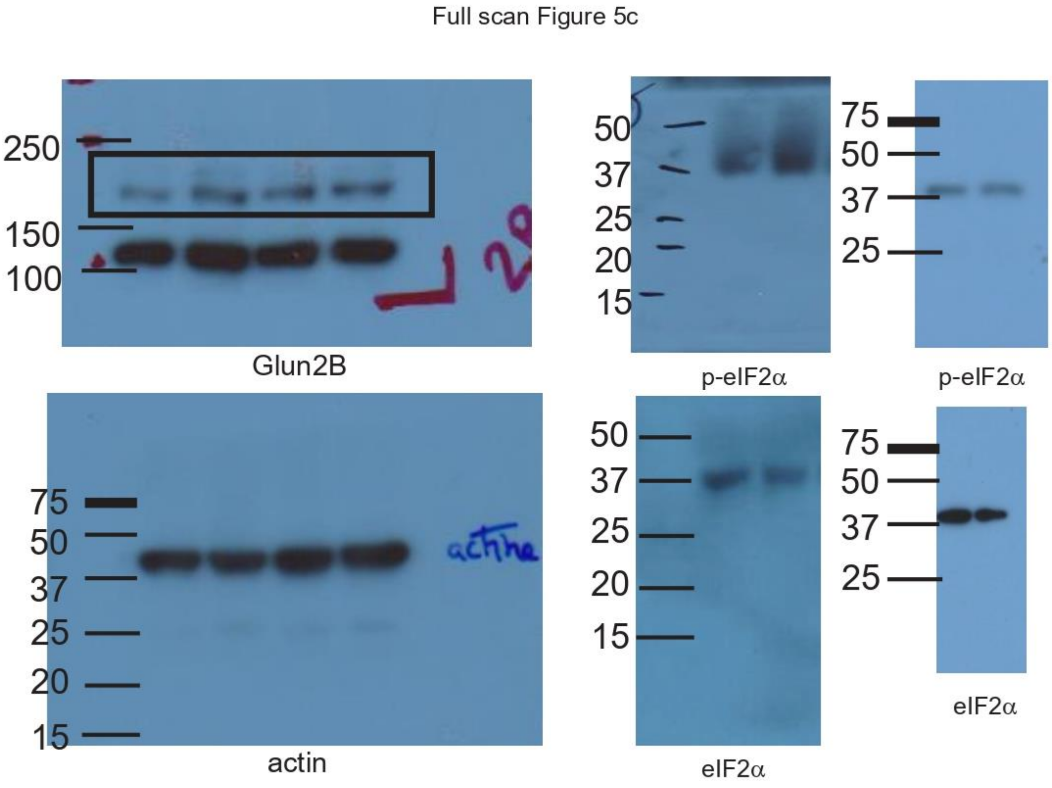

Figure S5. Full-length blots of Figure 5

Figure S6

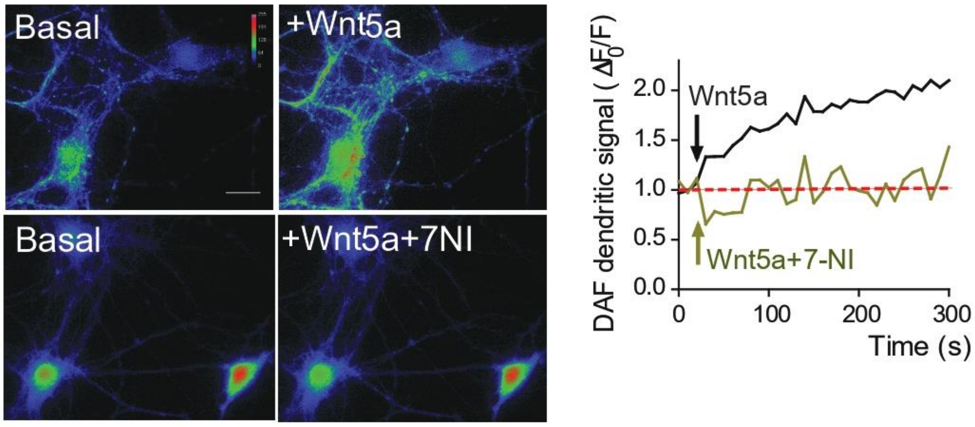

Figure S6. Wnt5a induces the production of NO. *a) Representative images and graph showing dendritic DAF fluorescent signal before and after Wnt5a application. Hippocampal neurons were incubated with the NO molecular probe DAF-FM. An immediate increase in DAF fluorescence was observed after Wnt5a application (right and left panels). This effect was severely prevented by the addition of 7-NI, a specific inhibitor of NO synthase (NOS). N=13-9 (dendrites). Scale bar: 10 µm.*

Figure S7

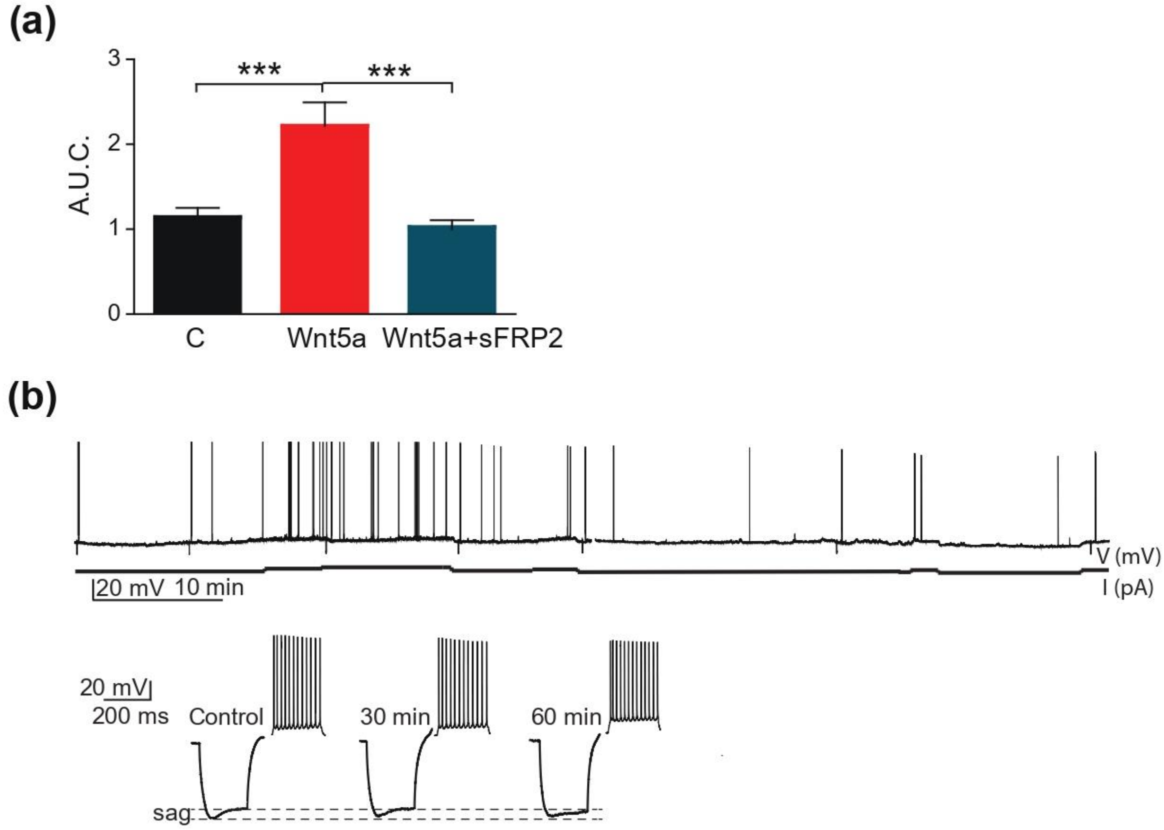

Figure S7. Calcium experiments of hippocampal neurons co incubated with sFRP2 and electrophysiological recordings in current clamp to demonstrate stability of long-term recordings. *a) Graphs depict the normalized AUC of the Fura-2 AM fluorescence ratio (340/380) after NMDA +glycine exposure in hippocampal neurons treated for 1 h with control (C) or Wnt5a (W5a) conditioned medium and/or sFRP2. N=5 independent experiments. C (100), Wnt5a (110), Wnt5a+sFRP2 (111) (***: p= 0.0001). b) Current-clamp recording of a CA1 pyramidal cell, showing the membrane potentials with spontaneous APs generated when purposely apply current through the circuit. Small deflections toward hyperpolarization indicate negative current pulses to determine* R_{in}*, which also evidence the sag component, a characteristic of h-current present in hippocampal pyramidal cells. The presence of sag as well as the APs amplitude and number after 1 h of recording, are an indication of healthy cell.*

Figure S8

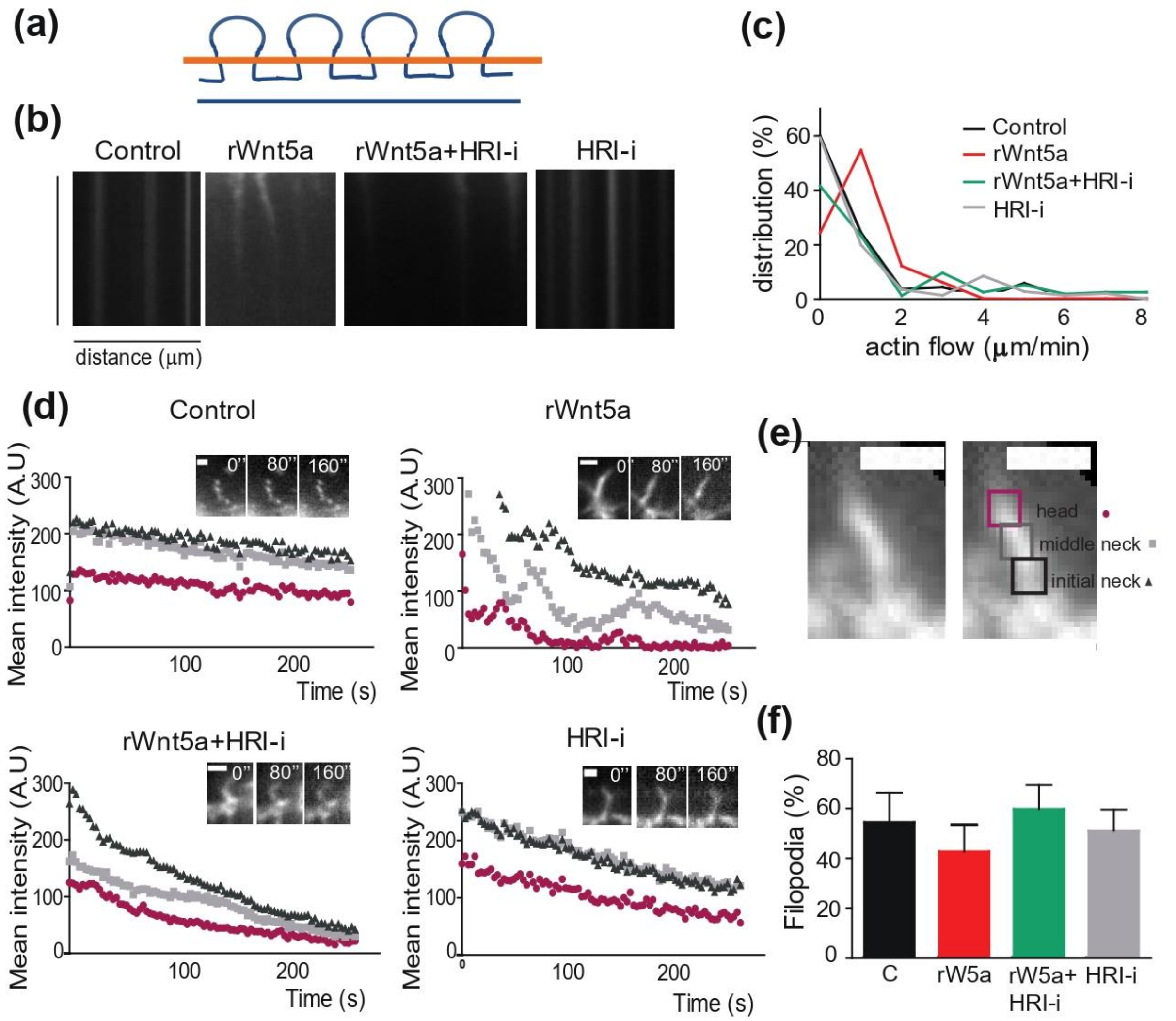

Figure S8. EGFP-LifeAct dynamics. *Kymographs were performed from at least 5 different ROIS (scheme included in (a)) from each video (b). (c) Histogram showing the % distribution of LifeAct-GFP positive protrusions vs the actin flow (µm/min). n=7 (control), n=12 (rWnt5a), n=7 (rWnt5a+HRI-i), n=5 (HRI-i) number of cells from 3 independent experiments. (d) Fluorescence intensity vs time graphs of each condition and insets showing representative protrusions. Scale bar: 1 µm (e) Scheme showing the regions selected for analysis of the distribution of fluorescence (data in d). Scale bar: 1 µm (f) Graph showing the % of filopodia in each condition. N=6 (control), N=8 (rWnt5a), N=6 (rWnt5a+HRI-i), N=6 (HRI-i) number of cells from 3 independent experiments.*

Supplementary videos legend

Video 1 (control), 2 (rWnt5a), 3 (rWnt5a+HRI-i) and 4 (HRI-i). *Movies of EGFP-LifeAct-transfected hippocampal neurons recorded each 3 sec during 1:30 min after 1 h with different treatments.*
